# Trends in Use of Medication to Treat Opioid Use Disorder During the COVID-19 Pandemic in 10 State Medicaid Programs

**DOI:** 10.1001/jamahealthforum.2023.1422

**Published:** 2023-06-16

**Authors:** Anna E. Austin, Lu Tang, Joo Yeon Kim, Lindsay Allen, Andrew J. Barnes, Chung-Chou H. Chang, Sarah Clark, Evan S. Cole, Christine Piette Durrance, Julie M. Donohue, Adam J. Gordon, Haiden A. Huskamp, Mary Joan McDuffie, Ateev Mehrotra, Shamis Mohamoud, Jeffery Talbert, Katherine A. Ahrens, Mary Applegate, Lindsey R. Hammerslag, Paul Lanier, Krystel Tossone, Kara Zivin, Marguerite E. Burns

**Affiliations:** 1 Department of Maternal and Child Health, Gillings School of Global Public Health, The University of North Carolina at Chapel Hill; 2 Injury Prevention Research Center, The University of North Carolina at Chapel Hill; 3 Department of Biostatistics, School of Public Health, University of Pittsburgh, Pittsburgh, Pennsylvania; 4 Department of Health Policy and Management, School of Public Health, University of Pittsburgh, Pittsburgh, Pennsylvania; 5 Department of Emergency Medicine, Feinberg School of Medicine, Northwestern University, Chicago, Illinois; 6 Department of Health Behavior and Policy, Virginia Commonwealth University, Richmond; 7 Department of Pediatrics, Michigan Medicine, University of Michigan, Ann Arbor; 8 La Follette School of Public Affairs, University of Wisconsin-Madison; 9 Department of Internal Medicine, University of Utah, Salt Lake City; 10 Department of Health Care Policy, Harvard Medical School, Boston, Massachusetts; 11 Center for Community Research and Service, Joseph R. Biden, Jr. School of Public Policy and Administration, University of Delaware, Newark; 12 The Hilltop Institute, University of Maryland, Baltimore County, Baltimore; 13 Institute for Biomedical Informatics, University of Kentucky, Lexington; 14 Public Health Program, Muskie School of Public Service, University of Southern Maine, Portland; 15 Ohio Department of Medicaid, Columbus; 16 School of Social Work, The University of North Carolina at Chapel Hill; 17 The Ohio Colleges of Medicine, Government Resource Center, College of Medicine, The Ohio State University, Columbus; 18 Department of Psychiatry, Michigan Medicine, University of Michigan, Ann Arbor; 19 Population Health Sciences, School of Medicine and Public Health, University of Wisconsin-Madison

## Abstract

**Importance:**

Federal and state agencies granted temporary regulatory waivers to prevent disruptions in access to medication for opioid use disorder (MOUD) during the COVID-19 pandemic, including expanding access to telehealth for MOUD. Little is known about changes in MOUD receipt and initiation among Medicaid enrollees during the pandemic.

**Objectives:**

To examine changes in receipt of any MOUD, initiation of MOUD (in-person vs telehealth), and the proportion of days covered (PDC) with MOUD after initiation from before to after declaration of the COVID-19 public health emergency (PHE).

**Design, Setting, and Participants:**

This serial cross-sectional study included Medicaid enrollees aged 18 to 64 years in 10 states from May 2019 through December 2020. Analyses were conducted from January through March 2022.

**Exposures:**

Ten months before the COVID-19 PHE (May 2019 through February 2020) vs 10 months after the PHE was declared (March through December 2020).

**Main Outcomes and Measures:**

Primary outcomes included receipt of any MOUD and outpatient initiation of MOUD via prescriptions and office- or facility-based administrations. Secondary outcomes included in-person vs telehealth MOUD initiation and PDC with MOUD after initiation.

**Results:**

Among a total of 8 167 497 Medicaid enrollees before the PHE and 8 181 144 after the PHE, 58.6% were female in both periods and most enrollees were aged 21 to 34 years (40.1% before the PHE; 40.7% after the PHE). Monthly rates of MOUD initiation, representing 7% to 10% of all MOUD receipt, decreased immediately after the PHE primarily due to reductions in in-person initiations (from 231.3 per 100 000 enrollees in March 2020 to 171.8 per 100 000 enrollees in April 2020) that were partially offset by increases in telehealth initiations (from 5.6 per 100 000 enrollees in March 2020 to 21.1 per 100 000 enrollees in April 2020). Mean monthly PDC with MOUD in the 90 days after initiation decreased after the PHE (from 64.5% in March 2020 to 59.5% in September 2020). In adjusted analyses, there was no immediate change (odds ratio [OR], 1.01; 95% CI, 1.00-1.01) or change in the trend (OR, 1.00; 95% CI, 1.00-1.01) in the likelihood of receipt of any MOUD after the PHE compared with before the PHE. There was an immediate decrease in the likelihood of outpatient MOUD initiation (OR, 0.90; 95% CI, 0.85-0.96) and no change in the trend in the likelihood of outpatient MOUD initiation (OR, 0.99; 95% CI, 0.98-1.00) after the PHE compared with before the PHE.

**Conclusions and Relevance:**

In this cross-sectional study of Medicaid enrollees, the likelihood of receipt of any MOUD was stable from May 2019 through December 2020 despite concerns about potential COVID-19 pandemic–related disruptions in care. However, immediately after the PHE was declared, there was a reduction in overall MOUD initiations, including a reduction in in-person MOUD initiations that was only partially offset by increased use of telehealth.

## Key Points

**Question:**

Were there changes in receipt and initiation of medication for opioid use disorder (MOUD) among Medicaid enrollees in 10 states from before to after declaration of the COVID-19 public health emergency (PHE)?

**Findings:**

In this cross-sectional study of 8 167 497 pre-PHE and 8 181 144 post-PHE Medicaid enrollees, receipt of any MOUD was stable from before to after declaration of the PHE. There was a reduction in overall MOUD initiations, primarily due to a reduction in in-person initiations that was only partially offset by an increase in telehealth initiations.

**Meaning:**

The results suggest that temporary regulatory waivers and expanded reimbursement for telehealth at the onset of the COVID-19 pandemic may have facilitated continued access to MOUD.

## Teaser

This cross-sectional study of Medicaid enrollees in 10 states assesses trends in use of medication for opioid use disorder (OUD) from before until after declaration of the COVID-19 public health emergency.

## Introduction

In 2021, there were an estimated 80 816 opioid-related overdose deaths in the US, a 62% increase since 2019.^1^ Early in the COVID-19 pandemic, there were large decreases in in-person health care visits,^2,3^ generating concerns regarding disruptions in access to medication for opioid use disorder (MOUD), evidence-based treatment for OUD.^4,5^ Compounding these concerns were the social and economic stressors of the pandemic, which had the potential to contribute to increased substance use,^4,5^ thus escalating overdose risk and demand for treatment.

Prior to the COVID-19 pandemic, federal laws and regulations required an in-person visit with a waivered prescriber for buprenorphine initiation and in-person opioid treatment program visits for methadone dispensing, with strict limits for take-home methadone.^6^ To prevent disruptions in access to MOUD during the pandemic, federal agencies implemented immediate and significant policy changes, including waiving requirements for initial in-person visits for buprenorphine prescribing and permitting an increased duration of take-home methadone.^7,8,9^ The Centers for Medicare & Medicaid Services also encouraged state Medicaid agencies to set reimbursement rates for telehealth visits at parity with in-person visits^10^ to increase use of telehealth, including for MOUD prescribing.

Recent studies provide insight into changes in MOUD access early in the pandemic. Using commercial insurance claims or retail pharmacy data, several studies found no change in MOUD receipt for existing patients but a decrease in new MOUD initiations after the COVID-19 public health emergency (PHE) was declared.^11,12,13^ Using Medicaid State Drug Utilization Data, another study reported that trends in buprenorphine prescriptions initially decreased after the PHE and then plateaued at pre-PHE levels in subsequent months.^14^ Importantly, that study was not conducted at the person level and did not examine other types of MOUD (methadone and naltrexone). In addition, the contribution of telehealth to changes in MOUD initiation among enrollees in Medicaid, which finances a large share of OUD treatment,^15^ is unknown.

The primary aims of this study were to examine changes in receipt of any MOUD and outpatient initiation of MOUD in the 10 months after the COVID-19 PHE was declared compared with the 10 months before the COVID-19 PHE was declared in 10 state Medicaid programs representing 22% of all Medicaid enrollees. The secondary aims were to examine changes in MOUD initiation via in-person or telehealth visits and the proportion of days covered (PDC) with MOUD after initiation.

## Methods

### Data Source

This cross-sectional study used data from the following 10 states participating in the Medicaid Outcomes Distributed Research Network (MODRN): Kentucky, Maine, Maryland, Michigan, North Carolina, Ohio, Pennsylvania, Virginia, West Virginia, and Wisconsin (eTable 5 in Supplement 1 lists the MODRN collaborators). Eight of these states had Medicaid expansion at the time of the study. University partners obtained data on a census of Medicaid enrollees from state Medicaid agencies and converted Medicaid claims and enrollment data to a common data model with uniform structure and data fields. A data coordinating center distributed standardized statistical code to states to apply to their common data model. Each state submitted aggregate results to the data coordinating center to combine for pooled analyses. Further details are available elsewhere.^16^ This study followed the Strengthening the Reporting of Observational Studies in Epidemiology (STROBE) reporting guideline and was considered exempt by participating university institutional review boards, with a waiver of informed consent because of use of deidentified secondary data.

### Study Population and Study Period

The study population included individuals aged 18 to 64 years who were enrolled in Medicaid at any point from May 2019 through December 2020 and were not dually eligible for Medicare (eTable 1 in Supplement 1). We defined the pre–PHE declaration period as May 2019 through February 2020 and the post–PHE declaration period as March 2020 through December 2020.

### Primary Outcome Measures

We constructed 2 primary outcome measures at the enrollee-month level: receipt of any MOUD and outpatient initiation of MOUD. The denominator for both outcomes included members of the study population enrolled in Medicaid in each month. We classified enrollees as receiving MOUD in a given month if they had an OUD diagnosis (*International Statistical Classification of Diseases, Tenth Revision, Clinical Modification* code F11.xxx) at some point during the study period and at least 1 claim with a National Drug Code for buprenorphine, buprenorphine/naloxone, or oral or injectable naltrexone in pharmacy claims or at least 1 outpatient claim with a Healthcare Common Procedure Coding System code for buprenorphine, buprenorphine/naloxone, or methadone administration in that month (eMethods 1 in Supplement 1). In 1 state, methadone was not covered by Medicaid during the first 2 months of the pre-PHE period (ie, May and June 2019).

We defined outpatient initiation of MOUD as a new treatment episode following 30 days^17^ enrolled in Medicaid with no days’ supply for MOUD prescriptions and no claims for office- or facility-based MOUD administration. Enrollees also had to have an OUD diagnosis at some point during the study period. We identified outpatient visits using Place of Service and *Current Procedural Terminology* codes, Healthcare Common Procedure Coding System codes included in our MOUD definition, and state-specific telehealth codes (eMethods 2 in Supplement 1). We focused on outpatient initiations only because inpatient and emergency department initiations could not be consistently and reliably measured across states. Enrollees could contribute more than 1 outpatient MOUD initiation during the study period.

### Secondary Outcomes

We also constructed 2 secondary outcome measures: visit modality for MOUD initiation and PDC with MOUD after initiation. We classified MOUD initiations by facility- and office-based administration as in-person initiations. To classify visit modality associated with MOUD initiations by prescription fill, we identified outpatient visits that occurred between 7 days before and 3 days after (−7/+3 days) the prescription fill.^18^ We considered visits in the −7/+3-day window to be associated with the MOUD prescription fill if there was a match between the prescriber identifier for the prescription and the clinician identifier for the visit or if there was an OUD diagnosis on the visit claim.

We are not aware of a validated approach to measuring telehealth in Medicaid claims. To identify telehealth initiations, we applied state-specific definitions obtained from each state Medicaid agency (eMethods 3 in Supplement 1). We conducted supplemental analyses using a previously published definition (eMethods 4 in Supplement 1).^2,18^ If any outpatient visits in the −7/+3-day window meeting our criteria were in-person, we considered the initiation to be in-person.^18^ If no in-person visits occurred during the −7/+3-day window and 1 or more telehealth visits were observed, we classified the initiation as telehealth. If no in-person or telehealth visits occurred in the −7/+3-day window, we considered the MOUD initiation to be unclassified (eFigure 1 in Supplement 1).

We calculated PDC with MOUD in the 90 days following initiation using prescription fill dates and days’ supply from pharmacy claims and service begin and end dates for office- and facility-based administrations. We assumed a 30-day supply for injectable naltrexone. We assigned PDC to the month of initiation and examined only MOUD initiations that occurred from May 2019 through September 2020 to ensure 90 days of follow-up for all initiations.

### Enrollee Characteristics and Commodities

We created the following indicators of enrollee characteristics using Medicaid enrollment data: sex (female or male), race and ethnicity as self-reported during the Medicaid enrollment process using fixed categories (Black non-Hispanic; Hispanic; White non-Hispanic; other racial and ethnic groups, including Alaska Native, American Indian, Asian, Native Hawaiian, and Pacific Islander; or unknown or missing), age group (18-20, 21-34, 35-44, 45-54, or 55-64 years), living area (rural or urban, defined from residence zip codes using rural-urban commuting area codes),^19^ Medicaid eligibility group (individuals with a disability, Medicaid expansion adults, youths aged 18-20 years, or adults without a disability or pregnant adults), and number of months enrolled in Medicaid during the study period. We constructed age group, living area, and eligibility group as time-varying indicators. We created the following indicators for comorbidities diagnosed during the study period: infectious diseases (HIV infection or hepatitis C or B), mental health conditions, non-OUD substance use disorders, and medical complications of injection drug use (intracranial or intraspinal abscess, osteomyelitis, endocarditis, or skin or soft tissue infection).

### Statistical Analysis

We pooled aggregate data on enrollee characteristics and comorbidities across all 10 states during the pre-PHE and post-PHE periods. We did not conduct statistical tests for comparisons as results were for the entire adult Medicaid population in these states, and we did not generalize results to all states.

We examined monthly trends in rates of each outcome overall and by MOUD type and modality of initiation. In supplemental analyses, we examined trends by state and enrollee characteristics.

To assess changes in our primary outcomes—any MOUD receipt and initiation of MOUD—in the post-PHE period compared with the pre-PHE period, we conducted interrupted time series analyses (eMethods 5 in Supplement 1). In these analyses, we specified the outcomes as binary indicators at the enrollee-month level and included indicators for the month from the start to the end of the study period to capture trends over time, the pre-PHE vs post-PHE period to capture the immediate change in the outcomes after the PHE, and the month from the start to the end of the post-PHE period to capture the change in the trend in the outcomes after the PHE. We used logistic regression to estimate odds ratios (ORs) and 95% CIs, adjusting for enrollee characteristics. We used generalized estimating equations to account for repeated measures within enrollees over time, specifying a first-order autoregressive working correlation matrix. We used 2 categories in multivariable models for the enrollee characteristics of race and ethnicity (minoritized racial and ethnic groups, White non-Hispanic) and Medicaid eligibility group (disability, nondisability) given small cell sizes and issues with model convergence in some states.

We conducted analyses from January through March 2022 in 2 stages, as is common in distributed research networks.^20^ First, each state conducted analyses using standardized statistical code developed by the data coordinating center. The data coordinating center then combined aggregate state-specific results. We used random effects meta-analysis to generate global ORs, averaging state-specific ORs weighted by the inverse of their variances.^20^ To describe heterogeneity in ORs across states, we calculated 90% prediction intervals (PIs), which indicate the range within which ORs would fall for 90% of states if we drew a different sample of states.^21^ We conducted meta-analyses in R, version 3.6.3 (R Project for Statistical Computing) using package metafor (3.0-2).

## Results

Among a total of 8 167 497 Medicaid enrollees before the PHE and 8 181 144 after the PHE, enrollee characteristics and comorbidities were similar in the pre-PHE and post-PHE periods (Table 1). Most enrollees were aged 21 to 34 years (40.1% before the PHE; 40.7% after the PHE). There was a slightly higher percentage of enrollees aged 18 to 20 years (10.6% vs 7.1%) and a lower percentage of enrollees aged 35 to 64 years (49.2% vs 52.2%) before the PHE compared with after the PHE. In both periods, 58.6% were female and 41.4% were male.

**Table 1.** Medicaid Enrollee Characteristics Before and After Declaration of the COVID-19 PHE^a^

Characteristic	Before the PHE (n = 8 167 497)		After the PHE (n = 8 181 144)	
	Medicaid enrollees, No. (%)	Range across states, %^b^	Medicaid enrollees, No. (%)	Range across states, %^b^
Age group, y				
18-20	867 899 (10.6)	9.3-20.7	580 312 (7.1)	5.6-13.3
21-34	3 277 289 (40.1)	36.6-42.0	3 327 263 (40.7)	38.0-42.3
35-44	1 710 936 (20.9)	18.7-23.7	1 793 979 (21.9)	20.3-24.4
45-54	1 297 986 (15.9)	12.3-17.3	1 335 423 (16.3)	12.4-18.5
55-64	1 013 387 (12.4)	10.4-14.4	1 144 167 (14.0)	12.1-16.3
Sex				
Female	4 788 731 (58.6)	56.5-67.5	4 790 465 (58.6)	55.7-69.9
Male	3 378 765 (41.4)	32.5-43.5	3 390 679 (41.4)	30.1-44.3
Race and ethnicity				
Black non-Hispanic	2 190 622 (26.8)	3.9-44.4	2 146 863 (26.2)	3.6-45.4
Hispanic	487 834 (6.0)	0.0-12.9	474 553 (5.8)	0.0-12.9
White non-Hispanic	4 523 866 (55.4)	30.7-84.5	4 547 627 (55.6)	30.6-84.3
Other racial and ethnic groups^c^	467 569 (5.7)	1.9-16.4	474 317 (5.8)	1.8-16.2
Unknown or missing	497 606 (6.1)	0.6-14.6	537 784 (6.6)	0.6-18.3
Medicaid eligibility group				
Individuals with a disability	1 033 121 (12.6)	2.2-24.3	1 021 332 (12.5)	2.5-24.4
Medicaid expansion adults	3 886 253 (47.6)	32.2-68.0	4 255 503 (52.0)	35.7-70.8
Youths aged 18-20 y	758 780 (9.3)	7.3-18.6	470 637 (5.8)	1.9-11.8
Adults without a disability or pregnant adults	2 489 343 (30.5)	17.9-57.0	2 433 672 (29.7)	15.6-63.8
Living area				
Urban	6 368 830 (78.0)	46.8-96.0	6 325 643 (77.3)	45.3-96.0
Rural	1 798 667 (22.0)	4.0-53.2	1 855 501 (22.7)	4.0-54.7
Comorbidities				
Infectious diseases^d^	270 668 (3.3)	1.6-4.9	268 789 (3.3)	1.6-5.0
Mental health conditions or other substance use disorders^e^	3 061 062 (37.5)	22.7-49.1	3 086 626 (37.7)	22.6-48.1
Medical complications of injection drug use^f^	756 306 (9.3)	4.7-12.3	753 777 (9.2)	4.6-12.5
Time enrolled in Medicaid during the study period, mean, mo	16.5	15.0-17.3	16.5	15.5-18.7

Abbreviation: PHE, public health emergency.

^a^ The period before the PHE was from May 2019 through February 2020; the period after the PHE was from March through December 2020.

^b^ Kentucky, Maine, Maryland, Michigan, North Carolina, Ohio, Pennsylvania, Virginia, West Virginia, and Wisconsin.

^c^ Includes Alaska Native, American Indian, Asian, Native Hawaiian, and Pacific Islander.

^d^ Infectious diseases include HIV infection or hepatitis C or B.

^e^ Anxiety, mood disorder, schizophrenia or other psychosis, posttraumatic stress disorder, or substance use disorders other than opioid use disorder and excluding tobacco use disorder.

^f^ Intracranial or intraspinal abscess, osteomyelitis, endocarditis, or skin or soft tissue infection.

### Unadjusted MOUD Outcomes

**Figure 1.**
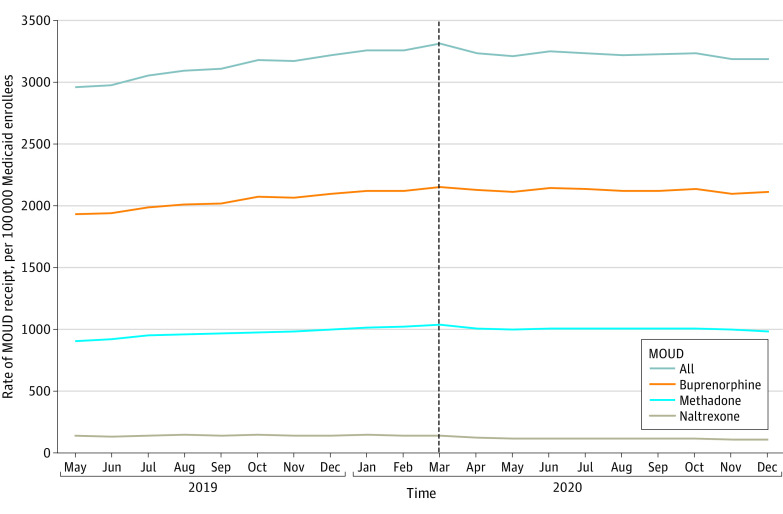
Monthly Rates of Any Receipt of Medication for Opioid Use Disorder (MOUD) Among Medicaid Enrollees in 10 States Medicaid enrollees were adults aged 18 to 64 years with full benefits who were not dually eligible for Medicare in Kentucky, Maine, Maryland, Michigan, North Carolina, Ohio, Pennsylvania, Virginia, West Virginia, and Wisconsin. Vertical dashed line indicates the time of declaration of the COVID-19 public health emergency.

The monthly rate of any MOUD receipt increased slightly throughout the pre-PHE period from 2996.2 per 100 000 enrollees in May 2019 to 3261.2 per 100 000 enrollees in February 2020 and was relatively stable in the post-PHE period (3312.0 per 100 000 enrollees in March 2020 and 3192.3 per 100 000 enrollees in December 2020) (Figure 1 and eTable 2 in Supplement 1). The monthly rate of buprenorphine receipt followed a similar trend. Monthly rates of methadone and naltrexone receipt were stable from before to after the PHE. By month, new MOUD initiations represented 7% to 10% of all MOUD administrations or prescription fills.

**Figure 2.**
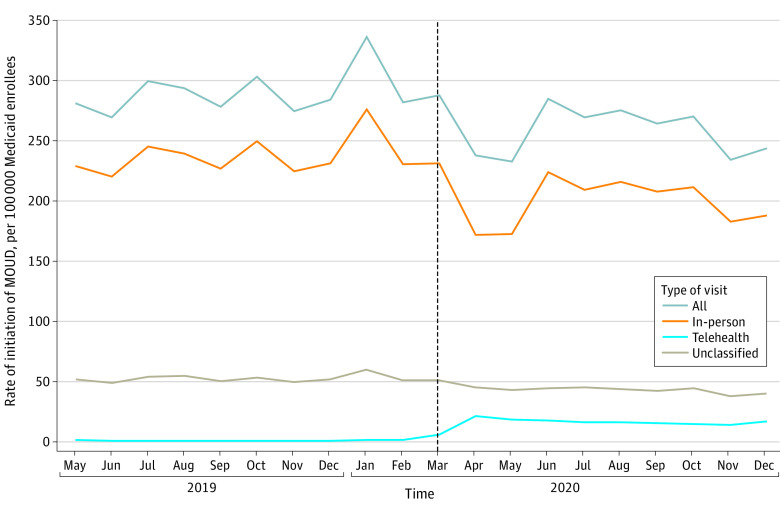
Monthly Rates of Outpatient Initiation of Medication for Opioid Use Disorder (MOUD) Among Medicaid Enrollees in 10 States Initiation was defined as a new MOUD treatment episode following 30 days with no MOUD. All office- and facility-based MOUD administrations were classified as in-person initiations. Telehealth vs outpatient initiation for prescription fills was determined by identifying outpatient visits within 7 days before to 3 days after the prescription fill with either a matching prescriber identification for the prescription and clinician identification for the visit or with an OUD diagnosis at the visit and then applying state-specific definitions for telehealth. MOUD prescription fills with no outpatient visits within 7 days before to 3 days after the prescription fill meeting these criteria were unclassified. Medicaid enrollees were adults aged 18 to 64 years with full benefits who were not dually eligible for Medicare in Kentucky, Maine, Maryland, Michigan, North Carolina, Ohio, Pennsylvania, Virginia, West Virginia, and Wisconsin. Vertical dashed line indicates the time of declaration of the COVID-19 public health emergency.

The monthly rate of overall MOUD initiations decreased immediately at the start of the post-PHE period from 288.0 per 100 000 enrollees in March 2020 to 237.7 per 100 000 enrollees in April 2020 and then increased beginning in June 2020 (to 285.2 per 100 000 enrollees) and decreased again beginning in November 2020 (to 234.5 per 100 000 enrollees) (Figure 2 and eTable 2 in Supplement 1). The monthly rate of initiations via in-person visits followed a similar trend, with a decrease at the start of the post-PHE period from 231.3 per 100 000 enrollees in March 2020 to 171.8 per 100 000 enrollees in April 2020.

The monthly rate of MOUD initiations via telehealth increased immediately at the start of the post-PHE period from 5.6 per 100 000 enrollees in March 2020 to 21.1 per 100 000 enrollees in April 2020 and remained relatively stable throughout the remainder of 2020. Telehealth accounted for 6% to 11% of all in-person and telehealth initiations per month from April through December 2020 compared with less than 1% before the PHE (eFigures 2 and 3 in Supplement 1). Monthly trends for MOUD initiations were similar using state-specific telehealth definitions or a common telehealth definition (eFigure 4 in Supplement 1).

Monthly rates of buprenorphine, methadone, and naltrexone initiations decreased immediately at the start of the post-PHE period from 185.7 per 100 000 enrollees in March 2020 to 162.6 per 100 000 enrollees in April 2020 for buprenorphine, from 63.3 per 100 000 enrollees in March 2020 to 43.4 per 100 000 enrollees in April 2020 for methadone, and from 39.0 per 100 000 enrollees in March 2020 to 31.7 per 100 000 enrollees in April 2020 for naltrexone (eFigure 5 in Supplement 1). Monthly rates of buprenorphine and methadone initiations increased somewhat in June 2020 (to 191.6 per 100 000 enrollees for buprenorphine and to 59.5 per 100 000 enrollees for methadone). Monthly rates of methadone initiations decreased again beginning in September 2020 (to 53.6 per 100 000 enrollees). Monthly rates of buprenorphine initiations decreased again in November 2020 (to 159.0 per 100 000 enrollees).

**Figure 3.**
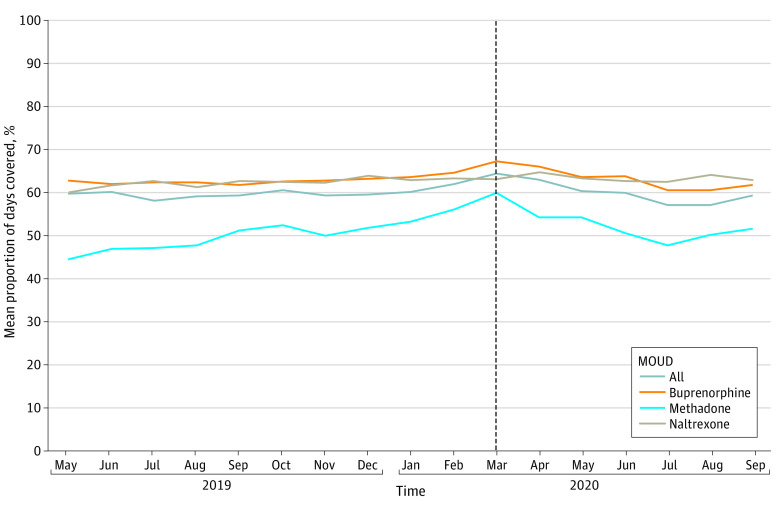
Monthly Mean Proportion of Days Covered With Medication for Opioid Use Disorder (MOUD) in the 90 Days After Initiation Among Medicaid Enrollees in 10 States Medicaid enrollees were adults aged 18 to 64 years with full benefits who were not dually eligible for Medicare in Kentucky, Maine, Maryland, Michigan, North Carolina, Ohio, Pennsylvania, Virginia, West Virginia, and Wisconsin. Mean proportion of days covered was calculated for initiations from May 2019 through September 2020 to ensure 90 days of follow-up in the data. Vertical dashed line indicates the time of declaration of the COVID-19 public health emergency.

Mean monthly PDC with MOUD in the 90 days after initiation decreased gradually at the start of the post-PHE period from 64.5% in March 2020 to 59.5% in December 2020 (Figure 3 and eTable 2 in Supplement 1). Mean monthly PDC with buprenorphine followed a similar trend. Mean monthly PDC with methadone decreased more quickly at the start of the post-PHE period. Mean monthly PDC with naltrexone did not change substantially.

For all outcomes, there was between-state variation in rates (eFigures 6-8 in Supplement 1). There were differences in rates of any MOUD receipt and outpatient MOUD initiation by enrollee sex, age group, race and ethnicity, and living area. These differences remained stable from the pre-PHE period to the post-PHE period (eFigures 9-16 in Supplement 1).

### Adjusted Changes in MOUD Outcomes Before vs After the PHE

When adjusting for enrollee characteristics, there was no immediate change in the likelihood of any MOUD receipt (OR, 1.01; 95% CI, 1.00-1.01) or in the trend in the likelihood of any MOUD receipt (OR, 1.00; 95% CI, 1.00-1.01) in the post-PHE period compared with the pre-PHE period (Table 2). The 90% PIs and state-specific results (eTable 3 in Supplement 1) indicated little heterogeneity across states.

**Table 2.** Interrupted Time Series Results for Change in the Likelihood of Any MOUD Receipt and Outpatient Initiation of MOUD After vs Before the COVID-19 PHE Declaration Among Medicaid Enrollees in 10 States^a^

Outcome	Average monthly percentage		Time trend			Immediate change after vs before the PHE			Trend change after vs before the PHE		
	Before the PHE	After the PHE	OR	95% CI	90% PI	OR	95% CI	90% PI	OR	95% CI	90% PI
Any MOUD receipt	3.13	3.23	1.01	1.00-1.01	0.99-1.03	1.01	1.00-1.01	0.99-1.02	1.00	1.00-1.01	0.99-1.01
Outpatient MOUD initiation	0.29	0.26	1.01	1.00-1.01	0.99-1.02	0.90	0.85-0.96	0.77-1.06	0.99	0.98-1.00	0.97-1.01

Abbreviations: MOUD, medication for opioid use disorder; OR, odds ratio; PHE, public health emergency; PI, prediction interval.

^a^ Adjusted for sex, race and ethnicity, age group, living area, Medicaid eligibility group, and number of months enrolled in Medicaid during the study period. Medicaid enrollees were adults aged 18 to 64 years with full benefits who were not dually eligible for Medicare in Kentucky, Maine, Maryland, Michigan, North Carolina, Ohio, Pennsylvania, Virginia, West Virginia, and Wisconsin. The period before the PHE was from May 2019 through February 2020; the period after the PHE was from March through December 2020.

There was an immediate decrease in the likelihood of outpatient MOUD initiation (OR, 0.90; 95% CI, 0.85-0.96) and no change in the trend in the likelihood of outpatient MOUD initiation (OR, 0.99; 95% CI, 0.98-1.00) in the post-PHE period compared with the pre-PHE period (Table 2). The 90% PIs and state-specific results (eTable 4 in Supplement 1) indicated some heterogeneity across states.

## Discussion

In this cross-sectional study of 10 state Medicaid programs, we found no substantial changes in overall rates of MOUD receipt in the post–COVID-19 PHE period compared with the pre–COVID-19 PHE period. We did, however, observe notable reductions in overall MOUD initiations in the post-PHE period. While MOUD initiations via telehealth increased after the PHE was declared, these increases were not large enough to offset reductions in in-person initiations.

Results showing no change in receipt of any MOUD from before to after the PHE declaration were consistent in meta-analyses and in state-specific analyses. This consistency across 10 distinct state Medicaid programs is notable given differences among states in overall rates of MOUD and COVID-19 pandemic responses. This finding suggests that the legal and regulatory shifts implemented to prevent pandemic-related disruptions in MOUD access may have been effective despite state variation in other policies and practices, although further analyses are needed. Prior studies also showed little change^22,23^ or a small slowing of prepandemic increases^14^ in rates of buprenorphine receipt early in the pandemic. We added to these studies by examining changes in all MOUD types combined and separately in 10 state Medicaid programs representing 22% of Medicaid enrollees.

Although we observed no substantial change in receipt of any MOUD, we observed an immediate decrease in overall MOUD initiations at the start of the post-PHE period. The immediate decrease in MOUD initiations after the PHE was declared was likely insufficient to alter the post-PHE trend in any MOUD receipt because MOUD initiations represented a small proportion of any MOUD receipt. In any given month, only 7% to 10% of MOUD prescription fills or administrations were initiations.

As overall and in-person MOUD initiations decreased after the PHE declaration, telehealth initiations increased. Telehealth initiations likely largely comprised buprenorphine initiations given that methadone and injectable naltrexone require office- or facility-based administration and thus cannot be initiated via telehealth. While we observed decreases in overall initiations of buprenorphine, methadone, and naltrexone in the initial months of the PHE, during which many states issued stay-at-home orders, the relative decrease was largest for methadone, perhaps because methadone cannot be initiated via telehealth.

By month, telehealth accounted for a higher percentage (6%-11%) of initiations from April through December 2020 compared with the pre-PHE period (<1%). These results are consistent with those of existing studies showing an increase in use of telehealth for other conditions,^2,11,18,24,25,26^ including among Medicaid enrollees, and for MOUD among Medicare beneficiaries^27^ during the pandemic. Of note, increases in MOUD initiations via telehealth at the start of the PHE may have helped to prevent even larger decreases in overall MOUD initiations than observed.

Mean PDC in the 90 days after initiation with buprenorphine decreased gradually in the post-PHE period, while mean monthly PDC with methadone decreased more quickly and to a greater degree. Changes to federal laws and regulations at the onset of the COVID-19 pandemic allowed for new initiations with buprenorphine via telehealth, but in-person evaluations were still required for methadone initiations.^28^ The decline in mean PDC with methadone at the start of the post-PHE period coincided with a sharp decline in in-person MOUD initiations. Telehealth restrictions for methadone initiation may have contributed to both a decline in initiation and to changes in the composition of patients initiating methadone that may have influenced PDC.

Temporary regulatory waivers and expanded reimbursement for telehealth at the onset of the COVID-19 pandemic^7,8,9,10^ may have facilitated continued access to MOUD for existing patients and were likely associated with increases in MOUD initiations via telehealth. There are calls to maintain these regulatory and policy shifts beyond the pandemic,^29,30,31^ particularly amid a worsening overdose crisis.^1^ While we cannot draw causal conclusions from our analyses regarding the effects of specific regulatory and policy shifts on MOUD access, recent research^32,33,34,35,36,37,38,39,40,41^ has highlighted the benefits and challenges to making these policy changes permanent.

Qualitative studies have demonstrated high patient and clinician satisfaction with telehealth for MOUD treatment during the pandemic,^32,33,34,35^ and studies have shown similar or improved outcomes associated with telehealth visits compared with in-person visits for MOUD treatment.^27,36,37,38^ Although clinicians largely report comfort in treating existing patients receiving MOUD via telehealth, many are hesitant to initiate MOUD via telehealth.^32,33,34^ For telehealth to remain a viable approach to delivering MOUD, additional clinical and policy guidance and continued parity for telehealth^34^ may be needed. Furthermore, some studies suggest differential use of telehealth by patient demographics, including race and ethnicity, age, and socioeconomic status.^39,40^ More evidence is needed on the potential for telehealth to either reduce or exacerbate existing inequities in MOUD access.^41^

### Limitations

This study has limitations. First, Medicaid claims data do not capture MOUD paid for by enrollees out of pocket. Whether out-of-pocket payments differed in the pre-PHE and post-PHE periods is unknown. Second, we required enrollees to have an OUD diagnosis during the study period to be classified as receiving MOUD or initiating MOUD. Codes in the *International Statistical Classification of Diseases, Tenth Revision, Clinical Modification* for OUD have limited sensitivity and specificity.^42,43^ In a previous study conducted within the MODRN, 87% of Medicaid enrollees receiving MOUD had claims with an OUD diagnosis.^20^ Third, we could not classify 16% to 18% of MOUD initiations per month as occurring in-person or via telehealth because there were no outpatient visits within the −7/+3-day window meeting our criteria. Fourth, early in the pandemic, clinicians may not have billed for all audio-only care, and we may have underestimated telehealth initiations during this time.^44^ Fifth, given that multiple events occurred within a short time frame (eg, onset of the COVID-19 pandemic, declaration of the PHE, maintenance of effort in Medicaid, and changes in reimbursement and regulatory policies), we could not determine which event(s) contributed to changes in MOUD.

## Conclusions

In this cross-sectional study, we found no substantial change in overall rates of MOUD from the pre-PHE to post-PHE period among Medicaid enrollees across 10 states. We also found a reduction in rates of overall MOUD initiations from the pre-PHE to post-PHE period. Although MOUD initiations via telehealth increased at the onset of the PHE, this increase was not sufficient to fully offset decreases in in-person MOUD initiations. Our results across 10 state Medicaid programs contribute valuable information to support ongoing discussions regarding the post–COVID-19 pandemic regulatory and payment environment that will best facilitate MOUD access.

## Notes

Supplement 1. **eTable 1.** Study Sample Construction Flowchart **eMethods 1.** Definition of Medication for Opioid Use Disorder (MOUD) **eMethods 2.** Definition of Outpatient Visits **eMethods 3.** State-Specific Definitions of Telehealth **eMethods 4.** Common Definition of Telehealth From Prior Research **eFigure 1.** Process for Classifying Medication for Opioid Use Disorder (MOUD) Initiations as In-Person, Telehealth, or Unclassified **eMethods 5.** Equation for Interrupted Time Series Models **eTable 2.** Medication for Opioid Use Disorder (MOUD) Outcomes Among Medicaid Enrollees in 10 States **eFigure 2.** Monthly Percentage of Outpatient Initiation of Medication for Opioid Use Disorder (MOUD) by Modality in 10 States **eFigure 3.** Monthly Rates of Outpatient Initiation of Medication for Opioid Use Disorder (MOUD) per 100,000 Medicaid Enrollees by Modality in 10 States **eFigure 4.** Monthly Rates of Outpatient Initiation of Medication for Opioid Use Disorder (MOUD) per 100,000 Medicaid Enrollees With a Common Definition for Telehealth in 10 States **eFigure 5.** Monthly Rates of Outpatient Initiation by Type of Medication for Opioid Use Disorder (MOUD) per 100 000 Medicaid Enrollees in 10 States **eFigure 6.** Monthly Rates of Any Medication for Opioid Use Disorder (MOUD) per 100 000 Medicaid Enrollees by State **eFigure 7.** Monthly Rates of Outpatient Initiation of Medication for Opioid Use Disorder (MOUD) per 100 000 Medicaid Enrollees by State **eFigure 8.** Monthly Mean Proportion of Days Covered With Medication for Opioid Use Disorder (MOUD) in the 90 Days After Initiation Among Medicaid Enrollees by State **eFigure 9.** Monthly Rates of Any Medication for Opioid Use Disorder (MOUD) per 100 000 Medicaid Enrollees by Age Group **eFigure 10.** Monthly Rates of Outpatient Initiation of Medication for Opioid Use Disorder (MOUD) per 100 000 Medicaid Enrollees by Age Group **eFigure 11.** Monthly Rates of Any Medication for Opioid Use Disorder (MOUD) per 100 000 Medicaid Enrollees by Race and Ethnicity **eFigure 12.** Monthly Rates of Outpatient Initiation of Medication for Opioid Use Disorder (MOUD) per 100 000 Medicaid Enrollees by Race and Ethnicity **eFigure 13.** Monthly Rates of Any Medication for Opioid Use Disorder (MOUD) per 100 000 Medicaid Enrollees by Sex **eFigure 14.** Monthly Rates of Outpatient Initiation of Medication for Opioid Use Disorder (MOUD) per 100 000 Medicaid Enrollees by Sex **eFigure 15.** Monthly Rates of Any Medication for Opioid Use Disorder (MOUD) per 100 000 Medicaid Enrollees by Living Area **eFigure 16.** Monthly Rates of Outpatient Initiation of Medication for Opioid Use Disorder (MOUD) per 100 000 Medicaid Enrollees by Living Area **eTable 3.** State-Specific Interrupted Time Series Results for Change in the Likelihood of Any Medication for Opioid Use Disorder (MOUD) After the COVID-19 Public Health Emergency Was Declared (Post-PHE) Compared With Before the Public Health Emergency Was Declared (Pre-PHE) Among Medicaid Enrollees in 10 States **eTable 4.** State-Specific Interrupted Time Series Results for Change in the Likelihood of Outpatient Initiation of Medication for Opioid Use Disorder (MOUD) After the COVID-19 Public Health Emergency Was Declared (Post-PHE) Compared With Before the Public Health Emergency Was Declared (Pre-PHE) Among Medicaid Enrollees in 10 States **eTable 5.** MODRN Collaborators

Supplement 2.
Data Sharing Statement
