# Supplementary material for: Trends in Use of Medication to Treat Opioid Use Disorder During the COVID-19 Pandemic in 10 State Medicaid Programs
Source: JAMA Health Forum. 2023 Jun 16;4(6):e231422. doi: 10.1001/jamahealthforum.2023.1422 (PMC10276306; doi:10.1001/jamahealthforum.2023.1422)
Supplement: Supplement 1. — eTable 1. Study Sample Construction Flowchart eMethods 1. Definition of Medication for Opioid Use Disorder (MOUD) eMethods 2. Definition of Outpatient Visits eMethods 3. State-Specific Definitions of Telehealth eMethods 4. Common Definition of Telehealth From Prior Research eFigure 1. Process for Classifying Medication for Opioid Use Disorder (MOUD) Initiations as In-Person, Telehealth, or Unclassified eMethods 5. Equation for Interrupted Time Series Models eTable 2. Medication for Opioid Use Disorder (MOUD) Outcomes Among Medicaid Enrollees in 10 States eFigure 2. Monthly Percentage of Outpatient Initiation of Medication for Opioid Use Disorder (MOUD) by Modality in 10 States eFigure 3. Monthly Rates of Outpatient Initiation of Medication for Opioid Use Disorder (MOUD) per 100,000 Medicaid Enrollees by Modality in 10 States eFigure 4. Monthly Rates of Outpatient Initiation of Medication for Opioid Use Disorder (MOUD) per 100,000 Medicaid Enrollees With a Common Definition for Telehealth in 10 States eFigure 5. Monthly Rates of Outpatient Initiation by Type of Medication for Opioid Use Disorder (MOUD) per 100 000 Medicaid Enrollees in 10 States eFigure 6. Monthly Rates of Any Medication for Opioid Use Disorder (MOUD) per 100 000 Medicaid Enrollees by State eFigure 7. Monthly Rates of Outpatient Initiation of Medication for Opioid Use Disorder (MOUD) per 100 000 Medicaid Enrollees by State eFigure 8. Monthly Mean Proportion of Days Covered With Medication for Opioid Use Disorder (MOUD) in the 90 Days After Initiation Among Medicaid Enrollees by State eFigure 9. Monthly Rates of Any Medication for Opioid Use Disorder (MOUD) per 100 000 Medicaid Enrollees by Age Group eFigure 10. Monthly Rates of Outpatient Initiation of Medication for Opioid Use Disorder (MOUD) per 100 000 Medicaid Enrollees by Age Group eFigure 11. Monthly Rates of Any Medication for Opioid Use Disorder (MOUD) per 100 000 Medicaid Enrollees by Race and Ethnicity eFigure 12. Monthly Rates of Outpatien [file jamahealthforum-e231422-s001.pdf]

Supplemental Online Content

Austin AE, Tang L, Kim JY, et al. Trends in use of medication to treat opioid use disorder during the COVID-19 pandemic in 10 state Medicaid programs. *JAMA Health Forum*. 2023;4(6):e231422. doi:10.1001/jamahealthforum.2023.1422

eTable 1. Study Sample Construction Flowchart
eMethods 1. Definition of Medication for Opioid Use Disorder (MOUD)
eMethods 2. Definition of Outpatient Visits
eMethods 3. State-Specific Definitions of Telehealth
eMethods 4. Common Definition of Telehealth From Prior Research
eFigure 1. Process for Classifying Medication for Opioid Use Disorder (MOUD) Initiations as In-Person, Telehealth, or Unclassified
eMethods 5. Equation for Interrupted Time Series Models
eTable 2. Medication for Opioid Use Disorder (MOUD) Outcomes Among Medicaid Enrollees in 10 States
eFigure 2. Monthly Percentage of Outpatient Initiations of Medication for Opioid Use Disorder (MOUD) by Modality in 10 States
eFigure 3. Monthly Rates of Outpatient Initiation of Medication for Opioid Use Disorder (MOUD) per 100,000 Medicaid Enrollees by Modality in 10 States
eFigure 4. Monthly Rates of Outpatient Initiation of Medication for Opioid Use Disorder (MOUD) per 100,000 Medicaid Enrollees With a Common Definition for Telehealth in 10 States
eFigure 5. Monthly Rates of Outpatient Initiation of Medication for Opioid Use Disorder (MOUD) per 100,000 Medicaid Enrollees by Type of MOUD in 10 States
eFigure 6. Monthly Rates of Any Medication for Opioid Use Disorder (MOUD) per 100,000 Medicaid Enrollees by State
eFigure 7. Monthly Rates of Outpatient Initiation of Medication for Opioid Use Disorder (MOUD) per 100,000 Medicaid Enrollees by State
eFigure 8. Monthly Mean Proportion of Days Covered With Medication for Opioid Use Disorder (MOUD) in the 90 Days After Initiation Among Medicaid Enrollees by State
eFigure 9. Monthly Rates of Any Medication for Opioid Use Disorder (MOUD) per 100,000 Medicaid Enrollees by Age Group
eFigure 10. Monthly Rates of Outpatient Initiation of Medication for Opioid Use Disorder (MOUD) per 100,000 Medicaid Enrollees by Age Group
eFigure 11. Monthly Rates of Any Medication for Opioid Use Disorder (MOUD) per 100,000 Medicaid Enrollees by Race and Ethnicity
eFigure 12. Monthly Rates of Outpatient Initiation of Medication for Opioid Use Disorder (MOUD) per 100,000 Medicaid Enrollees by Race and Ethnicity
eFigure 13. Monthly Rates of Any Medication for Opioid Use Disorder (MOUD) per 100,000 Medicaid Enrollees by Sex
eFigure 14. Monthly Rates of Outpatient Initiation of Medication for Opioid Use Disorder (MOUD) per 100,000 Medicaid Enrollees by Sex
eFigure 15. Monthly Rates of Any Medication for Opioid Use Disorder (MOUD) per 100,000 Medicaid Enrollees by Living Area
eFigure 16. Monthly Rates of Outpatient Initiation of Medication for Opioid Use Disorder (MOUD) per 100,000 Medicaid Enrollees by Living Area
eTable 3. State-Specific Interrupted Time Series Results for Change in the Likelihood of Any Medication for Opioid Use Disorder (MOUD) After the COVID-19 Public Health

© 2023 Austin AE et al. *JAMA Health Forum.*

Emergency Was Declared (Post-PHE) Compared With Before the Public Health Emergency Was Declared (Pre-PHE) Among Medicaid Enrollees in 10 States
eTable 4. State-Specific Interrupted Time Series Results for Change in the Likelihood of Outpatient Initiation of Medication for Opioid Use Disorder (MOUD) After the COVID-19 Public Health Emergency Was Declared (Post-PHE) Compared With Before the Public Health Emergency Was Declared (Pre-PHE) Among Medicaid Enrollees in 10 States
eTable 5. MODRN Collaborators

This supplemental material has been provided by the authors to give readers additional information about their work.

© 2023 Austin AE et al. *JAMA Health Forum.*

eTable 1. Study Sample Construction Flowchart

Description	
Number of member-months among non-dual, full benefit Medicaid enrollees between May 2019 - December 2020	345,676,285
Number of member-months excluding enrollees <18 years as of May 1, 2019 or ≥65 years as of December 31, 2020	141,379,843
Number of member-months excluding any monthly observation beyond month of date of death	141,254,246
Number of member-months excluding enrollees with missing urban/rural classification	140,950,666
Number of member-months read in models with the full cohort	140,950,666
Number of member-months used in models with the full cohort	140,950,664
Number of initiations	386,879

In Maine, Opioid Health Homes (OHHs) provide bundled treatment services for opioid use disorder. Medicaid enrollees in Maine who were Opioid Health Home (OHH) members were excluded.

© 2023 Austin AE et al. *JAMA Health Forum.*

eMethods1. Definition of Medication for Opioid Use Disorder (MOUD)

National Drug Code (NDC) in pharmacy claims for any of the following medications (oral and injectable):

Buprenorphine, naltrexone, buprenorphine/naloxone

or

Health Care common Procedure Coding System (HCPCS) code on an outpatient claim for any of the following medications:

Buprenorphine or buprenorphine/naloxone, oral: 'J0571', 'J0572', 'J0573', 'J0574', 'J0575'
Methadone administration: ‘H0020’
Naltrexone (extended-release injectable): ‘J2315’

Claims for oral medications with negative, missing, or zero days’ supply were not included.

© 2023 Austin AE et al. *JAMA Health Forum.*

eMethods 2. Definition of Outpatient Visits

Claims with any one of the following outpatient Current Procedural Terminology (CPT) and Health Care Common Procedure Coding System (HCPCS) codes:

00104 , 0901, 0911, 9424, 9426, 9427, 90791, 90792, 90801, 90802, 90804, 90805, 90806, 90807, 90808, 90809, 90810, 90811, 90812, 90813, 90814, 90815, 90820, 90832, 90833, 90834, 90835, 90836, 90837, 90838, 90839, 90840, 90842, 90843, 90844, 90845, 90846, 90847, 90848, 90849, 90853, 90855, 90857, 90862, 90865, 90867, 90868, 90869, 90870, 90871, 90875, 90876, 90880, 90900, 90901, 90902, 90904, 90906, 90908, 90910, 96372, 97003 , 97004, 98960, 98961, 98962, 99058, 99078, 99201, 99202, 99203, 99204, 99205, 99211, 99212, 99213, 99214, 99215, 99241, 99242, 99243, 99244, 99245, 99341, 99342, 99343, 99344, 99345, 99347, 99348, 99349, 99350, 99382, 99383, 99384, 99385, 99386, 99387, 99392, 99393, 99394, 99395, 99396, 99397, 99401, 99402, 99403, 99404, 99404, 99408, 99409, 99411, 99412, 99420, 99490, 99495, 99496, 99510, 0359T, 0360T, 0361T, 0362T, 0363T, 0364T, 0365T, 0366T, 0367T, 0368T, 0369T, 0370T, 0371T, 0372T, 0373T, 0374T, G0071, G0072, G0073, G0074, G0075, G0076, G0077, G0078, G0079, G0080, G0081, G0082, G0155, G0175, G0351, G0396, G0397, G0442, G0443, G0463, G0466, G0467, G0469, G0470, G0505, G0507, G0513, G0514, G0515, G2069, G2074, H0001, H0002, H0004, H0005, H0006, H0007, H0014, H0016, H0020, H0022, H0023, H0028, H0029, H0031, H0033, H0034, H0036, H0037, H0038, H0039, H0040, H0046, H0049, H0050, H1011, H2000, H2001, H2010, H2011, H2013, H2014, H2015, H2016, H2017, H2018, H2019, H2020, H2021, H2022, H2023, H2024, H2025, H2026, H2027, H2028, H2029, H2030, H2031, H2032, H2033, H2037, H5010, H5020, H5025, H5030, H5220, H5230, H5240, H5299, M0064, Q3014, S3005, S9110, S9127, S9454, S9482, S9484, S9485, T1006, T1006, T1007, T1011, T1012, T1015, T1016, T1017, T1018, T1023, T1024, T1025, T1026, T1027, T1040, T1041, T2010, T2011, T2012, T2013, T2014, T2015, T2018, T2019, T2020, T2021, T2022, T2023, T2034, T2036, T2037, Z0001, Z0002, Z0002

or

Claims any one of the following Place of Service (POS) codes:

02, 11, 12, 21, 22, 24, 25, 49, 50, 51, 55, 56, 61, 62, 65, 71, 15, 57, 58, 72

or

Claims with any one of the following HCPCS codes for medication for opioid use disorder:

'J0571', 'J0572', 'J0573', 'J0574', 'J0575', ‘H0020’, ‘J2315’

or

Claims with any one of the state-specific telehealth service codes (see Appendix Exhibit 4)

© 2023 Austin AE et al. *JAMA Health Forum.*

eMethods 3. State-Specific Definitions of Telehealth

Kentucky

- Modifier codes: ‘95’, ‘GT’, ‘GQ’
- Revenue code: ‘0780’
- POS code: ‘02’
- CPT/HCPCS codes: 'G2010’, ‘G2012’, ‘99421’, ‘99422’, ‘99423’, ‘99441’, ‘99442’, ‘99443’, ‘G2061’, ‘G2063’, ‘99457’, ‘99458’, ‘Q3014'
- CPT/HCPCS codes with modifier code ‘TG’ or ‘TF’: '97151’, ‘97153’, ‘97155’, ‘97156'
- CPT/HCPCS code ‘T1016’ with modifier code ‘TH’ or ‘95’
- CPT/HCPCS code ‘T1017’ with modifier code ‘U1’, ‘U2’, ‘U3’, ‘U4’, ‘U5’, ‘U6’
- CPT/HCPCS code ‘H0043’ with modifier code ‘U8’

Maine

- Modifier codes: ‘95’, ‘GT’
- POS code: ‘02’
- CPT/HCPCS codes: '99446', '99447', '99448', '99449', '99451', '99452', '0188T', '0189T', 'G0181', 'G0182', 'G0406', 'G0407', 'G0408', 'G0425', 'G0426', 'G0427', 'Q3014', 'T1014', 'S9110', 'G2010', 'G2012', 'G2061', 'G2062', 'G2063', 'G0071', '98966', '98967', '98968', '99421', '99422', '99423', '99441', '99442', '99443'

Maryland

For March 5, 2020-July 1, 2021”

- Modifier code:‘UB’

No time restriction:

- CPT/HCPCS code: ‘Q3014’
- Modifier code: ‘GT’

Michigan

No time restriction:

- CPT/HCPCS codes with modifier code ‘95’ or ‘GT’ or POS code ‘02’: '90785', '90791', '90792', '90832', '90833', '90834', '90836', '90837', '90838', '90839', '90840', '90846', '90847', '90849', '90853', '90863', '90887', '92507', '92508', '92521', '92522', '92523', '92524', '92626', '92627', '92630', '92633', '96103', '96105', '96110', '96112', '96113', '96116', '96120', '96121', '96127', '96130', '96131', '96132', '96133', '96136', '96137', '96138', '96139', '96146', '97110', '97112', '97116', '97161', '97162', '97163', '97164', '97165', '97166', '97167', '97168', '97530', '97535', '97760', '97761', '97763', '97802', '97803', '97804', '99201', '99202', '99203', '99204', '99205', '99211', '99212', '99213', '99214', '99215', '99221', '99222', '99223', '99224', '99225', '99226', '99231', '99232', '99233', '99307', '99308', '99309', '99310', '99324', '99325', '99326', '99327', '99328', '99334', '99335', '99336', '99337', '99341', '99342', '99343', '99344', '99345', '99347', '99348', '99349', '99350', '99605', 'G0176', 'G0177', 'G0409', 'G2067', 'G2068', 'G2073', 'G2074', 'G2076', 'G2077', 'G2080', 'H0001', 'H0002', 'H0004', 'H0005', 'H0015', 'H0022', 'H0023', 'H0025', 'H0031', 'H0032', 'H0034', 'H0036', 'H0038', 'H0039', 'H0043', 'H0046', 'H0050', 'H2010', 'H2011', 'H2014', 'H2015', 'H2019', 'H2021', 'H2022', 'H2023', 'H2027', 'H2030', 'H2033', 'S5110', 'S5111', 'S5116', 'S5140', 'S5145', 'S9445', 'S9446', 'S9470', 'S9482', 'S9484', 'T1001', 'T1002', 'T1012', 'T1015', 'T1016', 'T1017', 'T1023', 'T2015', 'T2023', 'T5999', '90951', '90952', '90954', '90955', '90957', '90958', '90960', '90961', '90963', '90964', '90965', '90966', '90967', '90968', '90969',

'90970', '92227', '92228', '96156', '96158', '96159', '96160', '96161', '96164', '96165', '96167', '96168', '99241', '99242', '99243', '99244', '99245', '99251', '99252', '99253', '99254', '99255', '99354', '99355', '99356', '99357', '99406', '99407', '99408', '99409', '99483', '99495', '99496', '99497', '99498', 'G0108', 'G0109', 'G0406', 'G0407', 'G0408', 'G0420', 'G0421', 'G0425', 'G0426', 'G0427', 'G0459', 'G0508', 'G0509', 'G2086', 'G2087', 'G2088', 'G2212', 'Q3014'

- Revenue codes with modifier code ‘95’ or ‘GT’ or POS code ‘02’: '0900', '0906', '0912', '0913', '0914', '0915', '0916', '0919'
- CPT/HCPCS codes with modifier code ‘HF’ AND (modifier code ‘95’ or ‘GT’ or POS code ‘02’): '99241', '99242', '99243', '99244', '99245', '99251', '99252', '99253', '99254', '99255', 'H2035', 'H2036', 'T1007'
- CPT/HCPCS code 'H2000' with modifier code ‘TS’ AND (modifier code ‘95’ or ‘GT’ or POS ‘02’)
- CPT/HCPCS codes with modifier code ‘U5’ AND (modifier code ‘95’ or ‘GT’ or POS ‘02’): '97151', '97153', '97154', '97155', '97156', '97157', '97158', '0362T'

On or after January 1, 2021:

- CPT/HCPCS codes with modifier code ‘95’ or ‘GT’ or POS code ‘02’: '99354', '99355', '99415', '99416', '99417'

North Carolina

Before January 1, 2020:

- CPT/HCPCS codes with modifier code ‘GT’: '99201', '99202', '99203', '99204', '99205', '99211', '99212', '99213', '99214', '99215', '99241', '99242', '99243', '99244', '99245', '99251', '99252', '99253', '99254', '99255', '99347', '99348', '99349', '99350', '99421', '99422', '99423', '99441', '99442', '99443', 'G2012', '99446', '99447', '99448', '99449', '99473', '99474', '99453', '99454', '99457', '99458'

On or after January 1, 2020:

- CPT/HCPCS codes with modifier code ‘GT’ and ‘CR’: '99201', '99202', '99203', '99204', '99205', '99211', '99212', '99213', '99214', '99215', '99241', '99242', '99243', '99244', '99245', '99251', '99252', '99253', '99254', '99255', '99347', '99348', '99349', '99350', '59400', '59410', '59425', '59426', '59430', '59510', '59515', '90832', '90834', '90837', '90846', '90847', '90951', '90952', '90953', '90954', '90955', '90956', '90957', '90958', '90959', '90960', '90961', '90962', '90963', '90964', '90965', '90966', '90967', '90968', '90969', '90970', '90989', '90993', '92065', '92507', '92521', '92522', '92523', '92524', '92526', '92607', '92608', '92609', '92630', '92633', '94010', '94060', '94150', '94375', '94664', '94760', '95992', '96110', '96112', '96113', '96116', '96121', '96125', '96130', '96131', '96132', '96133', '96136', '96137', '96138', '96139', '96146', '96156', '96158', '96159', '97110', '97112', '97116', '97151', '97152', '97153', '97154', '97155', '97161', '97162', '97163', '97164', '97165', '97166', '97167', '97168', '97530', '97533', '97535', '97542', '97750', '97763', '97802', '97803', '99201', '99202', '99203', '99204', '99205', '99211', '99212', '99213', '99214', '99215', '99231', '99232', '99233', '99238', '99239', '99241', '99242', '99243', '99244', '99245', '99307', '99308', '99309', '99310', '99406', '99407', '99503', '99504', 'H0031', 'H0036', 'T1017', 'T1023', 'G0108', 'H0038', 'H0040', 'H2011', 'H2022', 'H2033', 'Q3014', 'T1023'
- CPT/HCPCS codes with modifier code ‘CR’: '99421', '99422', '99423', '99441', '99442', '99443', 'G2012', '99446', '99447', '99448', '99449', '99473', '99474', '99453', '99454', '99457', '99458', '90785', '90791', '90792', '90832', '90833', '90834', '90836', '90837', '90838', '90839', '90840', '90846', '90847', '90849', '90853', '96127', '97156', '97157', '98966', '98967', '98968', 'G2012', 'H0010', 'H0012', 'H0013', 'H0015', 'H0019', 'H0035', 'H0038', 'H0040', 'H2011', 'H2017', 'H2020', 'H2022', 'H2033', 'H2035', 'H2036', 'RC0911', 'RC0183', 'S9484', 'T2013'

© 2023 Austin AE et al. *JAMA Health Forum.*

- CPT/HCPCS codes with modifier code ‘GT’ and ‘CR’ and POS ‘12’: '99501', '99502'
- CPT/HCPCS code ‘S9442’ with modifier code ‘GT’ and ‘CR’ and POS code ‘71’
- CPT/HCPCS codes with POS code ‘02’: 'D0140', 'D0170', 'D0999'
- CPT/HCPCS code ‘T2015’ with modifier code ‘GT’ and ‘CR’ and ‘HI’ and (POS code ‘50’ or ‘72’)
- CPT/HCPCS code ‘96161’ with modifier code ‘CR’ and ‘EP’
- CPT/HCPCS codes with modifier code ‘CR’ and ‘HA’: 'H2012', 'S9484'
- CPT/HCPCS code ‘H0028’ with modifier code ‘CR’ and ‘HQ’
- CPT/HCPCS code ‘H2015’ with modifier code ‘CR’ and ‘HT’ with modifier code: 'GT', 'HO', 'HF', 'HN', 'U1', 'HM'
- CPT/HCPCS code ‘H0036’ with modifier code ‘GT’ and ‘CR’ with modifier code: 'HI', 'HM', 'HQ', 'TL', 'UI'
- CPT/HCPCS codes with modifier code ‘GT’ or ‘CR’ or ‘FP’: '99201', '99202', '99203', '99204', '99205', '99211', '99213', '99214', '99215', '99241', '99242', '99243', '99244', '99441', '99442', '99443'
- CPT/HCPCS code 'S0280' or 'S0281'

No time restriction
- CPT/HCPCS codes 'T1015' with modifier code ‘GT’ and ‘CR’ and (POS code ‘50’ or ’72)
- CPT/HCPCS codes 'G0071' with modifier code ‘CR’ and (POS code ‘50’ or ’72)

Ohio
- Modifier codes: ‘95’, ‘GT’, ‘GQ’
- CPT/HCPCS codes: '99441', '99442', '99421', '99422', '99423', '99457', '99458'

Pennsylvania
- Modifier codes: ‘95’, ‘GT’, ‘CR’, ‘DR’
- POS code: ‘02’
- CPT/HCPCS codes: 'D9995' or 'Q3014'

Virginia
- CPT/HCPCS codes with modifier code ‘GT’: 'Q3014', '90833', '90834', '90836', '90837', '90838', '90846', '90847', '90853', '90863', '90791', '90792', '90785', '90832', 'T1012', 'S9445'

West Virginia
- Modifier codes: ‘GT’, ‘GY’
- Revenue code: ‘0780’, ‘0789’
- POS code: ‘02’
- CPT/HCPCS codes: 'D9995', '99444', '99421', '99422', '99423', 'G2061', 'G2062', 'G2063', 'G2010', 'G2012', 'Q3014', '98966', '98967', '98968', '99441', '99442', '99443'
- CPT/HCPCS codes with modifier code ‘95’ or ‘GT’: '77427', '90785', '90791', '90792', '90832', '90833', '90834', '90836', '90837', '90838', '90839', '90840', '90845', '90846', '90847', '90853', '90875', '90951', '90952', '90953', '90954', '90955', '90956', '90957', '90958', '90959', '90960', '90961', '90962', '90963', '90964', '90965', '90966', '90967', '90968', '90969', '90970', '92002', '92004', '92012', '92014', '92507', '92508', '92521', '92522', '92523', '92524', '92601', '92602', '92603', '92604', '93797', '93798', '93750', '94002', '94003', '94004', '94005', '94664', '95970', '95971', '95972', '95983', '95984', '96110', '96112', '96113', '96116', '96121', '96127', '96130', '96131', '96132', '96133', '96136', '96137', '96138', '96139', '96156', '96158', '96159', '96160', '96161', '96164', '96165', '96167', '96168', '96168', '96170', '96171', '97110', '97112', '97116', '97150', '97151', '97152', '97153', '97154', '97155', '97156', '97157', '97158', '97161', '97162',

'97163', '97164', '97165', '97166', '97167', '97168', '97530', '97535', '97542', '97750', '97755', '97760', '97761', '97802', '97803', '97804', '99202', '99203', '99204', '99205', '99211', '99212', '99213', '99214', '99215', '99217', '99218', '99219', '99220', '99221', '99222', '99223', '99224', '99225', '99226', '99231', '99232', '99233', '99234', '99235', '99236', '99238', '99239', '99281', '99282', '99283', '99284', '99285', '99291', '99292', '99304', '99305', '99306', '99307', '99308', '99309', '99310', '99315', '99316', '99324', '99325', '99326', '99327', '99328', '99334', '99335', '99336', '99337', '99341', '99342', '99343', '99344', '99345', '99347', '99348', '99349', '99350', '99354', '99355', '99356', '99357', '99406', '99407', '99441', '99442', '99443', '99468', '99469', '99471', '99472', '99473', '99475', '99476', '99477', '99478', '99479', '99480', '99483', '99495', '99496', '99497', '99498', '0373T', 'S9152', '0362T', 'G0108', 'G0109', 'G0270', 'G0296', 'G0396', 'G0397', 'G0406', 'G0407', 'G0408', 'G0410', 'G0420', 'G0421', 'G0422', 'G0423', 'G0424', 'G0425', 'G0426', 'G0427', 'G0438', 'G0439', 'G0442', 'G0443', 'G0444', 'G0445', 'G0446', 'G0447', 'G0459', 'G0506', 'G0508', 'G0509', 'G0513', 'G0514', 'G2086', 'G2087', 'G2088', 'G9685', 'G2211', 'G2212'

Wisconsin

Before September 1, 2020:

- CPT/HCPCS code '99201' with modifier code: ‘95’ or ‘GT’ or POS code ‘02’

On or after March 1, 2020:

- Modifier code ‘95’ or ‘GT’ or POS ‘02’
- CPT/HCPCS codes with modifier code ‘U8’: 'H0043', 'H0038'
- CPT/HCPCS codes with modifier code ‘TG’ or ‘TF’: '97151', '97153', '97155', '97156'

No time restriction

- CPT/HCPCS codes with modifier code ‘95’ or ‘GT’ or POS code ‘02’: 'H2012', '92550', '92585', '92586', '92587', '92588', 'H0039', 'H2017', 'S9484', '90951', '90952', '90954', '90955', '90956', '90957', '90958', '90960', '90961', '90967', '90968', '90969', '90970', '96156', '96158', '96159', '96167', '96170', '96171', '99251', '99252', '99253', '99254', '99255', '99202', '99203', '99204', '99205', '99211', '99212', '99213', '99214', '99215', '99241', '99242', '99243', '99244', '99245', '90785', '90791', '90792', '90832', '90833', '90834', '90836', '90837', '90838', '90839', '90840', '90845', '90846', '90847', '90849', '90875', '90876', '90887', 'H0022', 'H0047', 'T1006'

© 2023 Austin AE et al. *JAMA Health Forum.*

eMethods 4. Common Definition of Telehealth From Prior Research[1]

- POS code: ‘02’

or

- Modifier code: ‘GT’, ‘GQ’, ‘95’, ‘G0’

or

- HCPCS codes:
    - ‘G0425-7’ (telehealth consultations emergency department or initial inpatient)
    - ‘G2025’ (when a FQHC / Rural health clinic provides telemedicine services)
    - ‘G0406-8’ (follow-up inpatient or skilled nursing facility consultations via telehealth)
    - ‘G0459’ (pharmacologic management service furnished via telehealth to inpatients)
    - ‘G0508-9’ (telehealth consultations for critical care)
    - ‘0188T’ (remote real-time interactive video conferenced critical care, first 30- 74 minutes)
    - ‘99441’–‘99443’
    - ‘G2012’, ‘G2010’, ‘99421’-‘99423’, ‘G2061-3’, ‘G0071’, ‘G2250-2’, ‘98970-2’
      Note: ‘G2250-2’, ‘98970-2’ are new codes FY2021 that replace ‘G2010’/’G2012’, ‘G2061-3’
    - ‘99446’-‘99452’
    - ‘98966’–‘98968’
    - ‘99453’, ‘99454’, ‘99457’, ‘9909’1, ‘95250’, ‘95251’

Excluding HCPCS code ‘Q3014’ even if accompanied by one of the above POS or modifier codes

[1]Barsky BA, Busch AB, Patel SY, Mehrotra A, Huskamp HA. Use of Telemedicine for Buprenorphine Inductions in Patients With Commercial Insurance or Medicare Advantage. *JAMA Network Open*. 2022;5(1).

eFigure 1. Process for Classifying Medication for Opioid Use Disorder (MOUD) Initiations as In-Person, Telehealth, or Unclassified

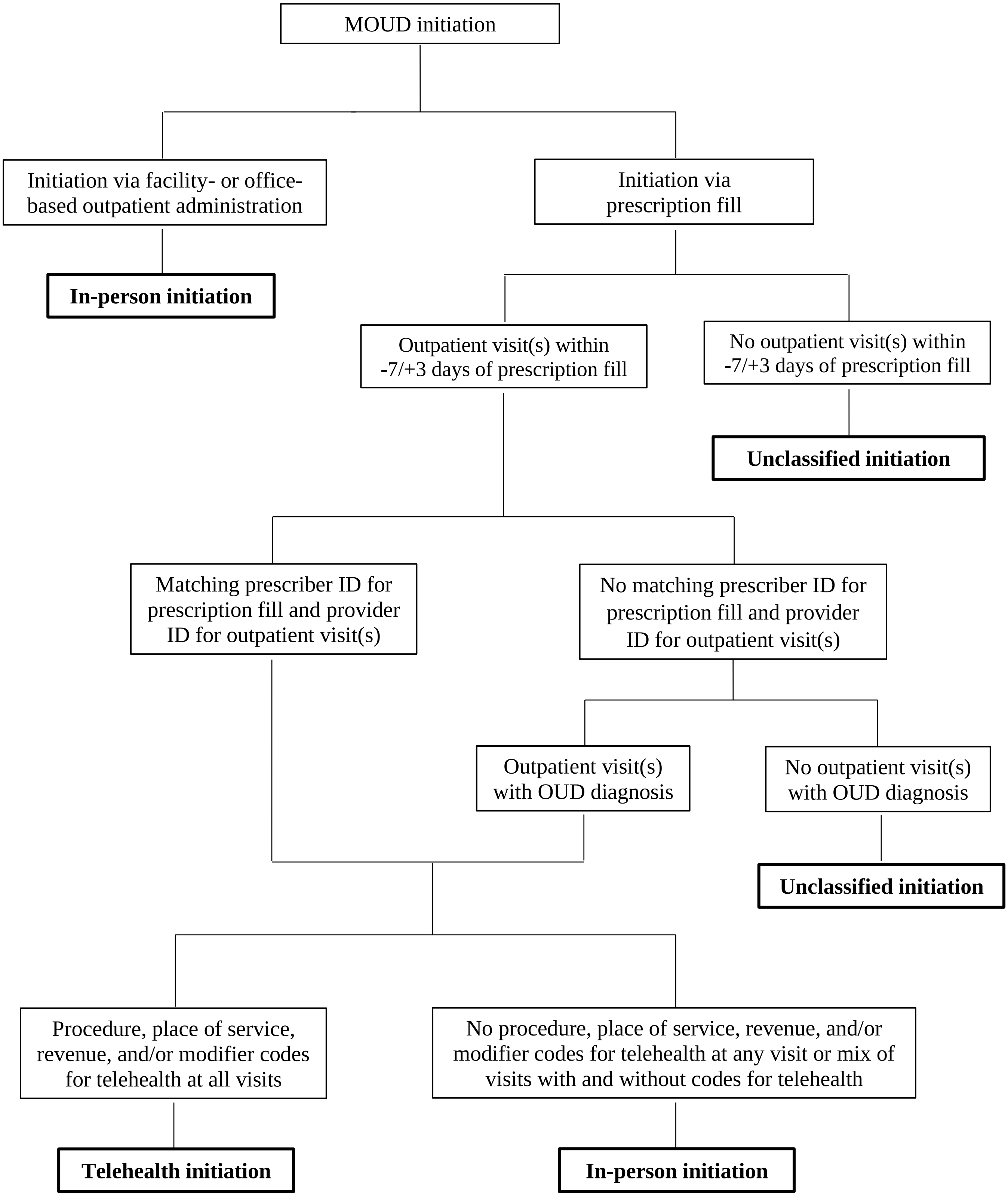

© 2023 Austin AE et al. *JAMA Health Forum.*

eMethods 5. Equation for Interrupted Time Series Models

To assess changes in any MOUD and outpatient initiation of MOUD in the post-PHE period relative to the pre-PHE period, we conducted interrupted time series (ITS) analyses at the enrollee-level, specifying mean models as follows:

$$g(E[Y_{it}]) = \beta_0 + \beta_1(time)_t + \beta_2(PHE)_t + \beta_3(Post\text{-}PHE\ time)_t + X_i\theta + P_{it}\sigma$$

where Y_{it} is a binary indicator for the outcome for enrollee *i* in month-year *t* and *g* is the logit link function defined as $g(p) = \ln(\frac{p}{1-p})$. *Time* is an integer variable indicating month from the start to the end of the study period. *PHE* is a binary variable equal to 1 in the post-PHE period (March 2020-December 2020). *Post-PHE time* is an integer variable indicating month from the start of the post-PHE period, where March 2020 is month 1. β_2 captures the immediate change in the outcome in the post-PHE period. β_3 captures the change in the trend in the outcome in the post-PHE period relative to the pre-PHE period. *X* is a vector of time-invariant enrollee characteristics, *P* is a vector of time-varying enrollee characteristics. We used generalized estimation equations with logit link to calculate odds ratios (ORs) and the robust covariance matrix estimator (i.e., sandwich estimator) to calculate the 95% confidence intervals (CIs), accounting for repeated measures within enrollees over time, specifying a first-order autoregressive correlation matrix.

© 2023 Austin AE et al. *JAMA Health Forum.*

eTable 2. Medication for Opioid Use Disorder (MOUD) Outcomes Among Medicaid Enrollees in 10 States[a]

Year and month	Rate of any MOUD per 100,000 enrollees				Rate of outpatient MOUD initiation[b] per 100,000 enrollees				Mean proportion of days covered with MOUD in the 90 days after initiation			
	All MOUD types	Buprenorphine	Methadone	Naltrexone	All initiations	In-person initiations	Telehealth initiations	Unclassified initiations	All MOUD types	Buprenorphine	Methadone	Naltrexone
2019-05	2,966.2	1,933.5	910.7	143.8	281.1	228.9	0.9	51.3	59.9	62.9	44.7	60.1
2019-06	2,980.5	1,943.3	924.9	134.4	269.2	220.4	0.5	48.3	60.3	62.1	47.1	61.8
2019-07	3,058.7	1,986.0	950.5	145.4	299.9	245.1	0.7	54.1	58.2	62.5	47.4	62.9
2019-08	3,098.3	2,015.7	960.2	146.8	294.1	239.2	0.8	54.2	59.3	62.5	48.0	61.4
2019-09	3,113.6	2,020.4	973.5	142.5	278.1	227.2	0.8	50.1	59.4	61.9	51.4	62.8
2019-10	3,182.6	2,073.3	981.5	152.9	303.7	250.0	0.8	52.9	60.8	62.7	52.6	62.6
2019-11	3,175.0	2,065.3	989.3	143.0	274.5	224.4	0.6	49.5	59.5	62.9	50.2	62.5
2019-12	3,218.8	2,098.2	1,002.8	142.4	284.2	231.5	0.8	51.9	59.6	63.4	51.9	64.1
2020-01	3,260.1	2,125.0	1,013.3	150.0	336.7	276.1	1.3	59.4	60.3	63.8	53.4	63.0
2020-02	3,261.2	2,122.9	1,023.9	140.5	282.2	230.3	1.4	50.6	62.2	64.7	56.3	63.4
2020-03	3,312.0	2,155.2	1,039.5	143.2	288.0	231.3	5.6	51.1	64.5	67.5	60.0	63.2
2020-04	3,239.2	2,127.5	1,008.0	127.7	237.7	171.8	21.1	44.7	63.2	66.1	54.4	64.8
2020-05	3,211.8	2,116.4	1,001.7	118.8	233.1	172.4	17.9	42.9	60.6	63.7	54.5	63.5
2020-06	3,252.7	2,147.1	1,010.6	121.8	285.2	224.2	17.1	43.9	60.1	64.0	50.8	62.9
2020-07	3,238.1	2,137.4	1,007.9	119.6	269.7	209.5	15.6	44.6	57.3	60.7	47.9	62.7
2020-08	3,225.2	2,121.3	1,012.2	118.3	275.6	216.2	16.1	43.2	57.3	60.7	50.4	64.3
2020-09	3,227.1	2,122.9	1,011.0	119.1	264.7	207.5	15.0	42.2	59.5	62.0	51.7	63.0
2020-10	3,240.1	2,135.5	1,013.2	118.7	270.0	211.2	14.4	44.4				
2020-11	3,192.0	2,101.4	1,003.6	111.8	234.5	182.9	13.7	37.9				
2020-12	3,192.3	2,118.8	985.2	112.4	243.8	187.8	16.4	39.7				

[a]Non-dual full benefit Medicaid enrollees ages 18-64 years in Kentucky, Maine, Maryland, Michigan, North Carolina, Ohio, Pennsylvania, Virginia, West Virginia, Wisconsin

[b]Initiation defined as a new MOUD treatment episode following 30 days with no MOUD. All office- and facility-based MOUD administrations were classified as in-person initiations. Telehealth vs. outpatient initiation for prescription fills was determined by identifying outpatient visits within 7 days before to 3 days after the prescription fill with either a matching prescriber ID for the prescription and provider ID for the visit or with an opioid use disorder diagnosis at the visit and then applying state-specific definitions for telehealth. MOUD prescription fills with no outpatient visits within 3 days prior to 7 days after the prescription fill meeting these criteria were unclassified.

Note: Mean proportion of days covered with MOUD in the 90 days after initiation calculated for May 2019-Septemter 2020 to ensure all initiations had 90 days of follow-up in our data.

© 2023 Austin AE et al. *JAMA Health Forum.*

eFigure 2. Monthly Percentage of Outpatient Initiation[a] of Medication for Opioid Use Disorder (MOUD) by Modality in 10 States[b]

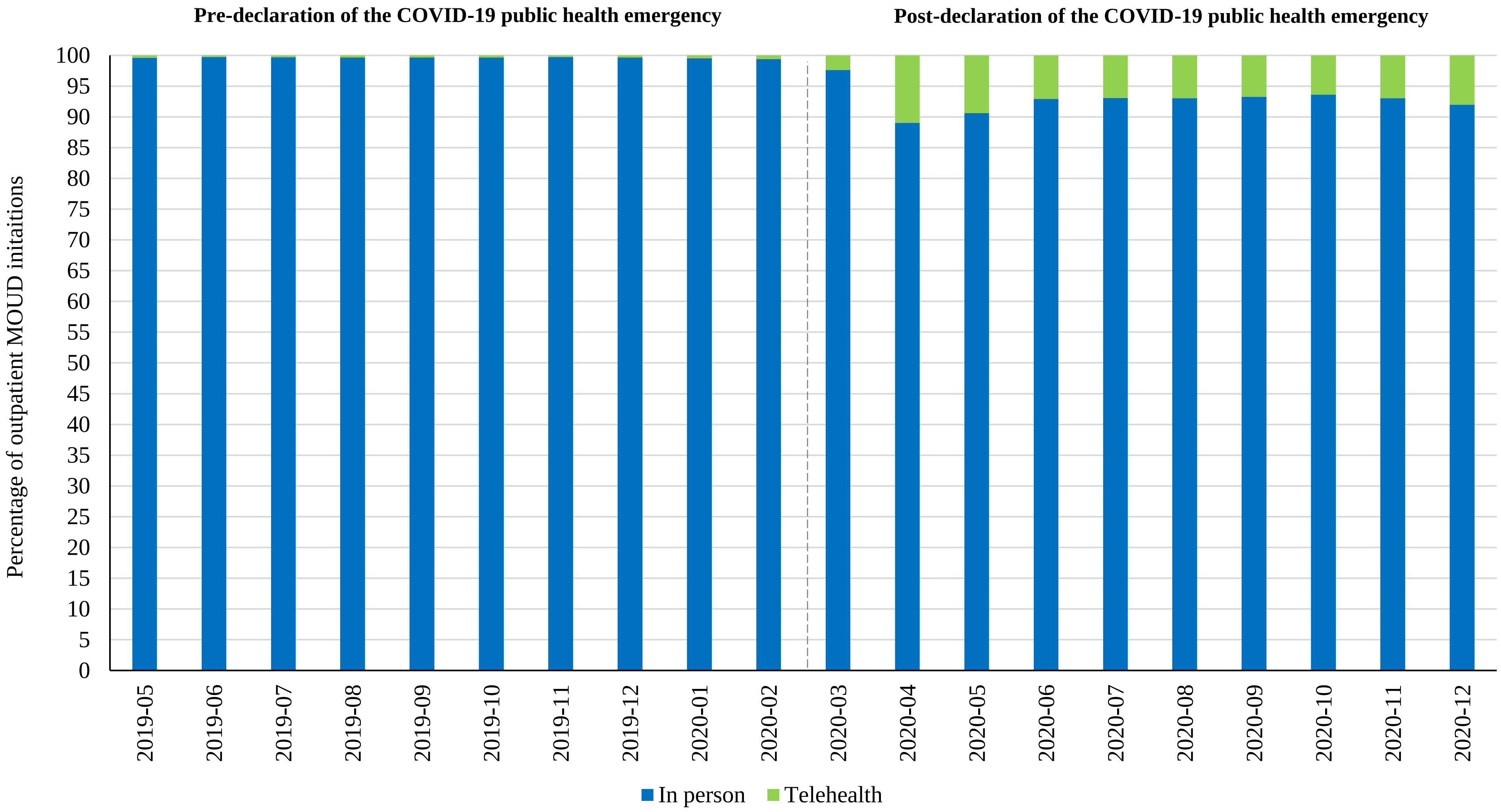

[a]Initiation defined as a new MOUD treatment episode following 30 days with no MOUD. All office- and facility-based MOUD administrations were classified as in-person initiations. Telehealth vs. outpatient initiation for prescription fills was determined by identifying outpatient visits within 7 days before to 3 days after the prescription fill with either a matching prescriber ID for the prescription and provider ID for the visit or with an opioid use disorder diagnosis at the visit and then applying state-specific definitions for telehealth. MOUD prescription fills with no outpatient visits within 7 days before to 3 days after the prescription fill meeting these criteria were unclassified.

[b]Non-dual full benefit Medicaid enrollees ages 18-64 years in Kentucky, Maine, Maryland, Michigan, North Carolina, Ohio, Pennsylvania, Virginia, West Virginia, Wisconsin

© 2023 Austin AE et al. *JAMA Health Forum.*

eFigure 3. Monthly Rates of Outpatient Initiation[a] of Medication for Opioid Use Disorder (MOUD) per 100,000 Medicaid Enrollees by Modality in 10 States[b]

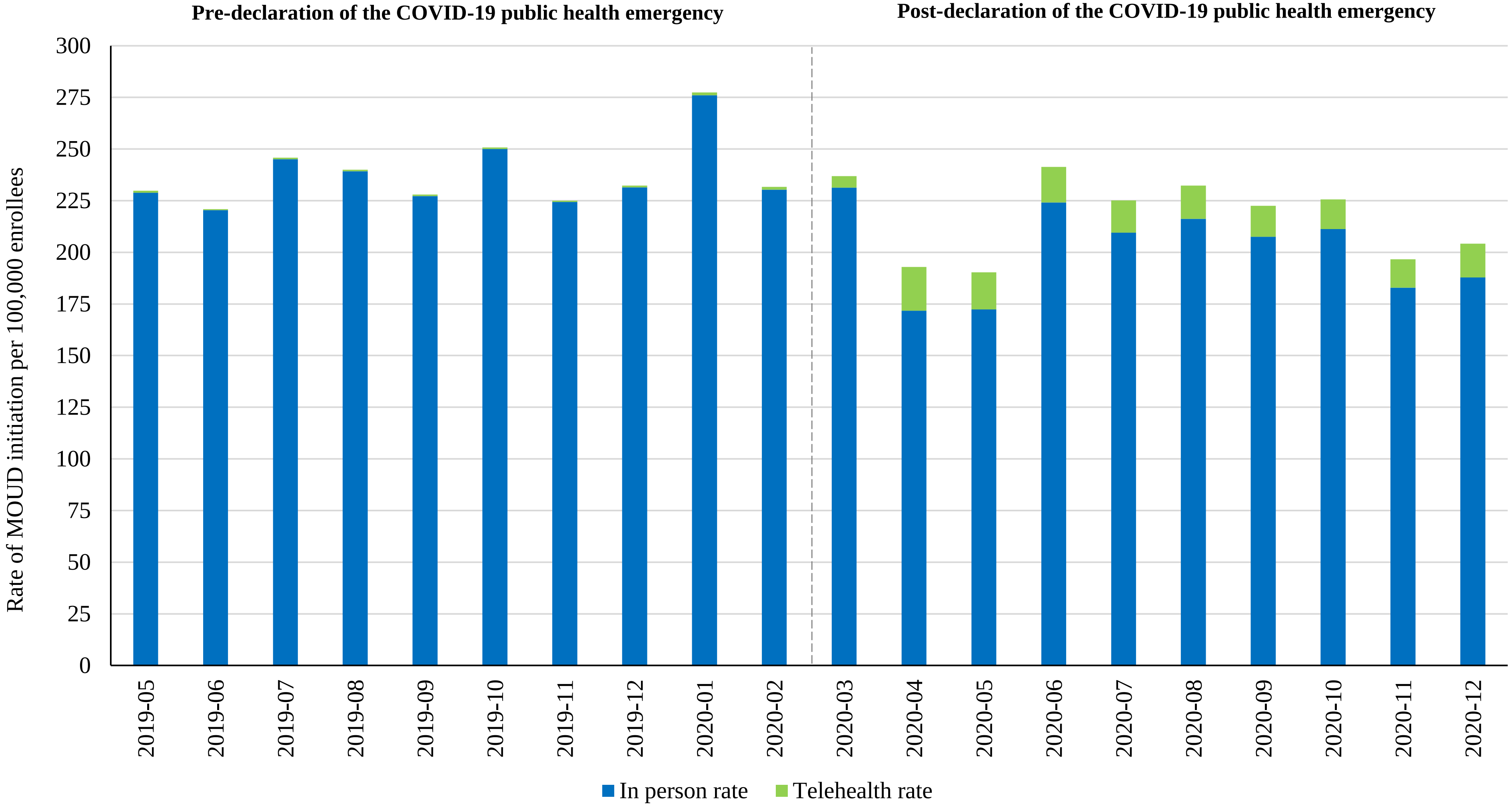

[a]Initiation defined as a new MOUD treatment episode following 30 days with no MOUD. All office- and facility-based MOUD administrations were classified as in-person initiations. Telehealth vs. outpatient initiation for prescription fills was determined by identifying outpatient visits within 7 days before to 3 days after the prescription fill with either a matching prescriber ID for the prescription and provider ID for the visit or with an opioid use disorder diagnosis at the visit and then applying state-specific definitions for telehealth. MOUD prescription fills with no outpatient visits within 7 days before to 3 days after the prescription fill meeting these criteria were unclassified.
[b]Non-dual full benefit Medicaid enrollees ages 18-64 years in Kentucky, Maine, Maryland, Michigan, North Carolina, Ohio, Pennsylvania, Virginia, West Virginia, Wisconsin

© 2023 Austin AE et al. *JAMA Health Forum.*

eFigure 4. Monthly Rates of Outpatient Initiation[a] Medication for Opioid Use Disorder (MOUD) per 100,000 Medicaid Enrollees With a Common Definition for Telehealth in 10 States[b]

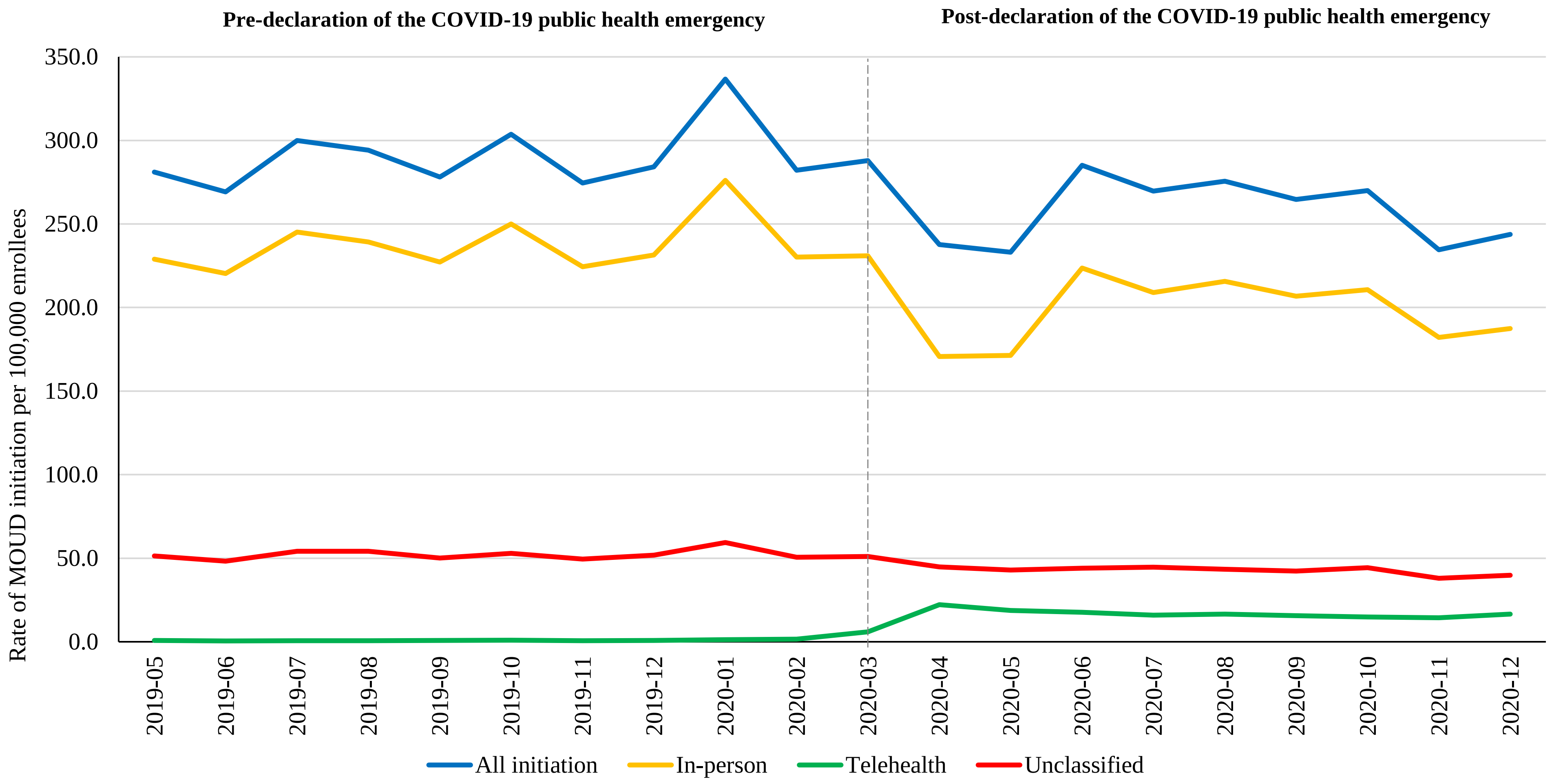

[a]Initiation defined as a new MOUD treatment episode following 30 days with no MOUD. All office- and facility-based MOUD administrations were classified as in-person initiations. Telehealth vs. outpatient initiation for prescription fills was determined by identifying outpatient visits within 7 days before to 3 days after the prescription fill with either a matching prescriber ID for the prescription and provider ID for the visit or with an opioid use disorder diagnosis at the visit and then applying state-specific definitions for telehealth. MOUD prescription fills with no outpatient visits within 7 days before to 3 days after the prescription fill meeting these criteria were unclassified.

© 2023 Austin AE et al. *JAMA Health Forum.*

[b]Non-dual full benefit Medicaid enrollees ages 18-64 years in Kentucky, Maine, Maryland, Michigan, North Carolina, Ohio, Pennsylvania, Virginia, West Virginia, Wisconsin

eFigure 5. Monthly Rates of Outpatient Initiation[a] of Medication for Opioid Use Disorder (MOUD) per 100,000 Medicaid Enrollees by Type of MOUD in 10 States[b]

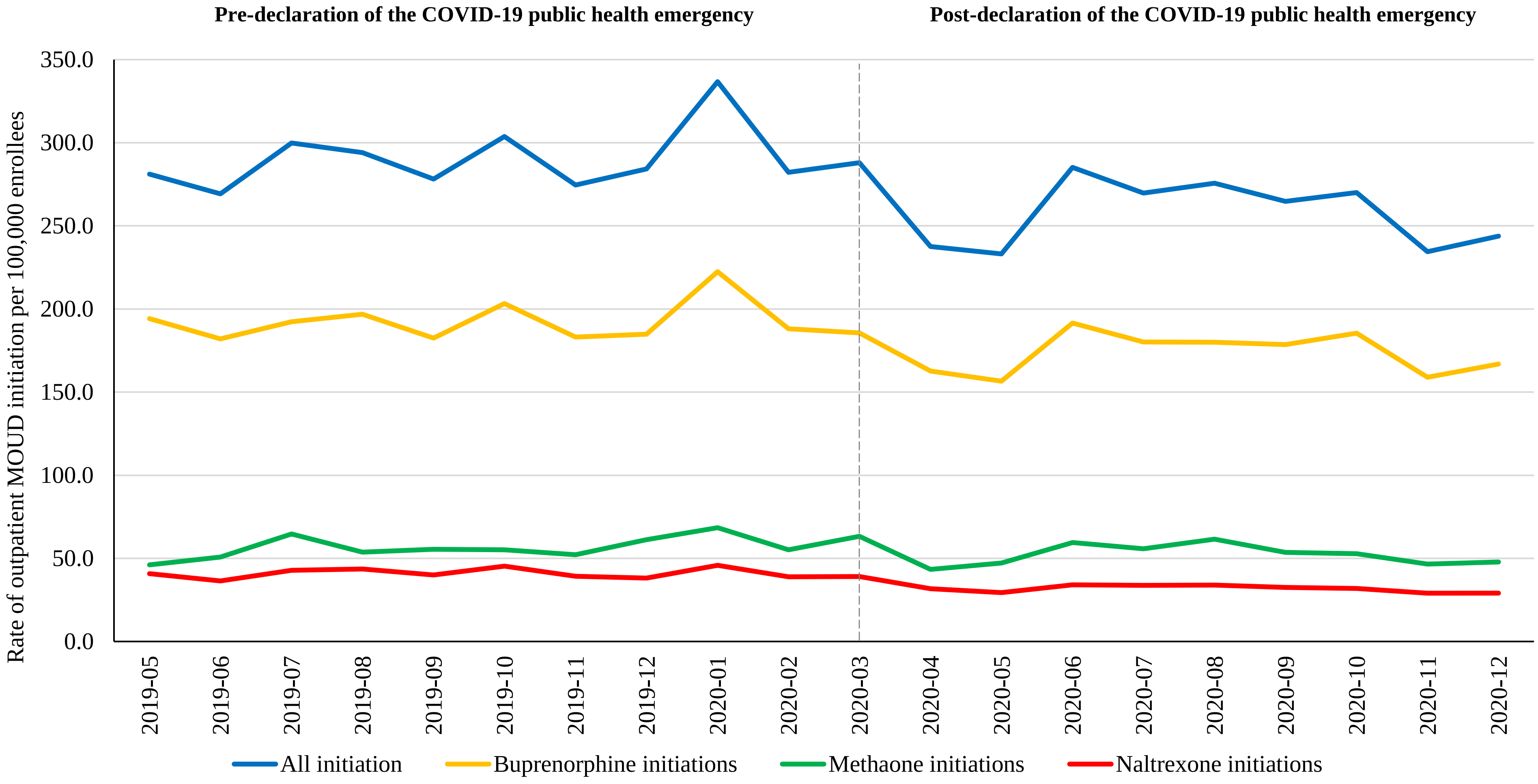

[a]Initiation defined as a new MOUD treatment episode following 30 days with no MOUD. All office- and facility-based MOUD administrations were classified as in-person

© 2023 Austin AE et al. *JAMA Health Forum.*

initiations. Telehealth vs. outpatient initiation for prescription fills was determined by identifying outpatient visits within 7 days before to 3 days after the prescription fill with either a matching prescriber ID for the prescription and provider ID for the visit or with an opioid use disorder diagnosis at the visit and then applying state-specific definitions for telehealth. MOUD prescription fills with no outpatient visits within 7 days before to 3 days after after the prescription fill meeting these criteria were unclassified.
[b]Non-dual full benefit Medicaid enrollees ages 18-64 years in Kentucky, Maine, Maryland, Michigan, North Carolina, Ohio, Pennsylvania, Virginia, West Virginia, Wisconsin

eFigure 6. Monthly Rates of Any Medication for Opioid Use Disorder (MOUD) per 100,000 Medicaid Enrollees by State[a]

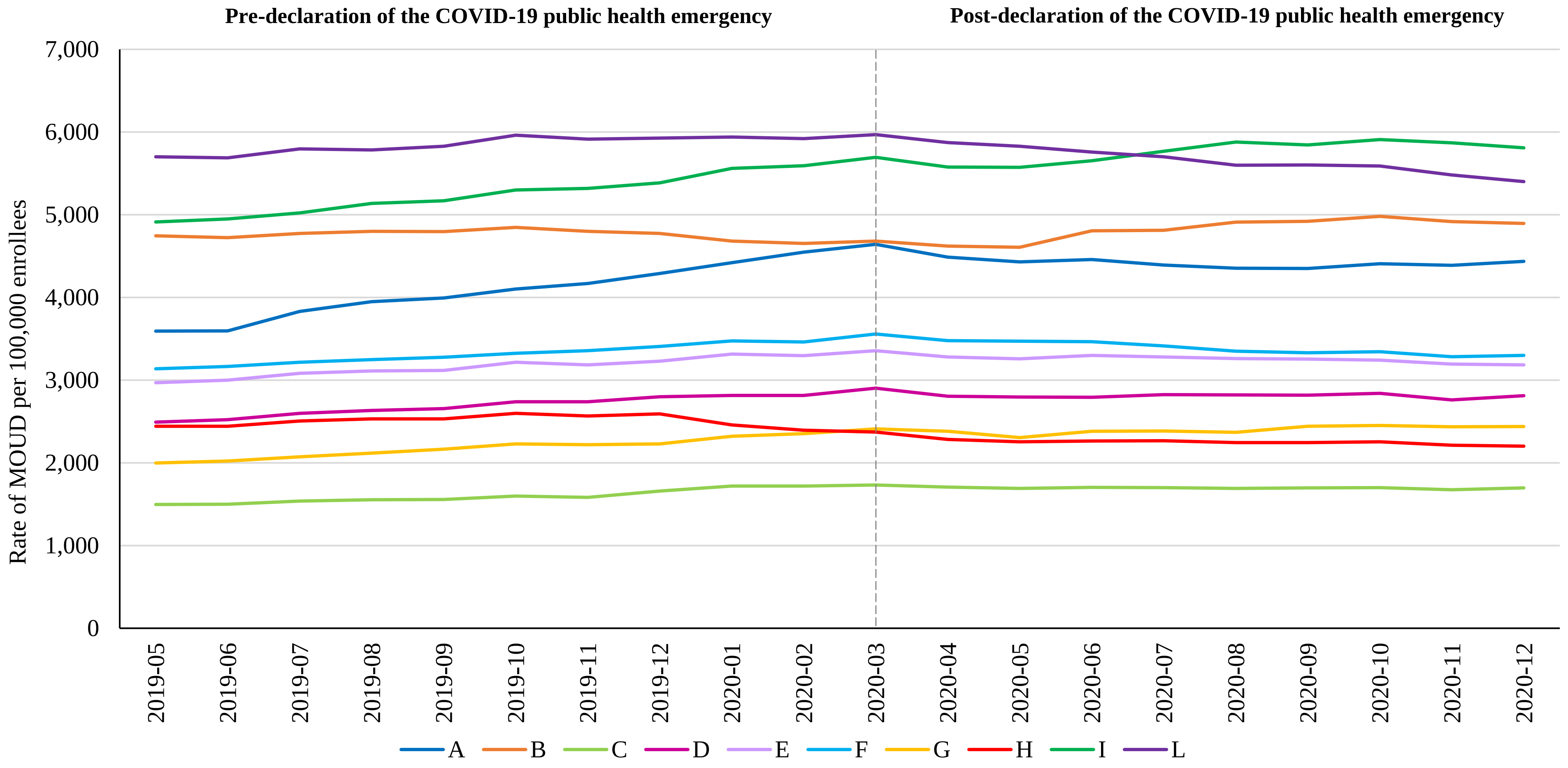

[a]Non-dual full benefit Medicaid enrollees ages 18-64 years in Kentucky, Maine, Maryland, Michigan, North Carolina, Ohio, Pennsylvania, Virginia, West Virginia, Wisconsin

© 2023 Austin AE et al. *JAMA Health Forum.*

eFigure 7. Monthly Rates of Outpatient Initiation[a] of Medication for Opioid Use Disorder (MOUD) per 100,000 Medicaid Enrollees by State[b]

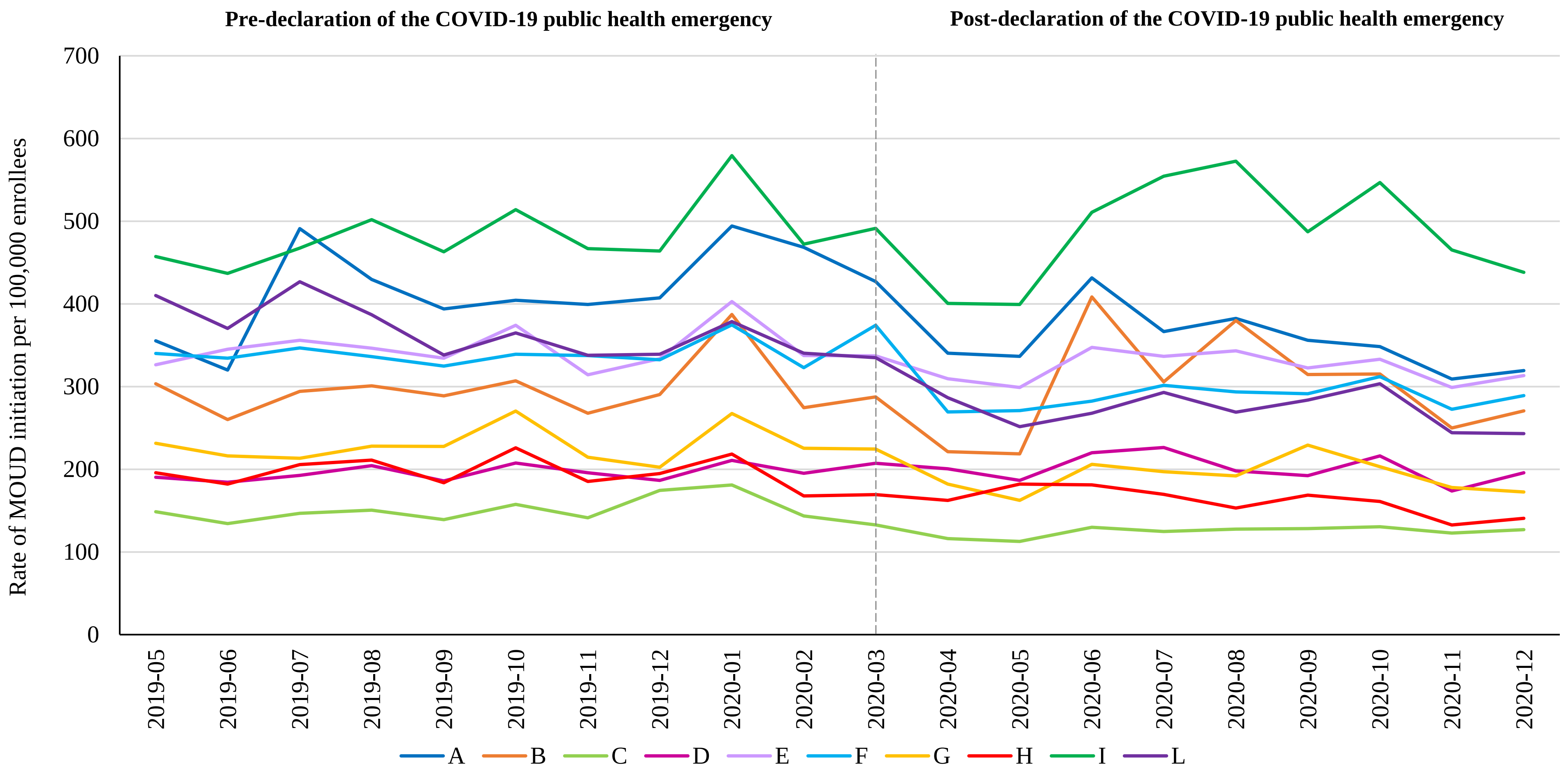

[a]Initiation defined as a new MOUD treatment episode following 30 days with no MOUD. All office- and facility-based MOUD administrations were classified as in-person initiations. Telehealth vs. outpatient initiation for prescription fills was determined by identifying outpatient visits within 7 days before to 3 days after the prescription fill with either a matching prescriber ID for the prescription and provider ID for the visit or with an opioid use disorder diagnosis at the visit and then applying state-specific definitions for telehealth. MOUD prescription fills with no outpatient visits within 7 days before to 3 days after the prescription fill meeting these criteria were unclassified.
[b]Non-dual full benefit Medicaid enrollees ages 18-64 years in Kentucky, Maine, Maryland, Michigan, North Carolina, Ohio, Pennsylvania, Virginia, West Virginia, Wisconsin

© 2023 Austin AE et al. *JAMA Health Forum.*

eFigure 8. Monthly Mean Proportion of Days Covered With Medication for Opioid Use Disorder (MOUD) in the 90 Days After Initiation Among Medicaid Enrollees by State[a]

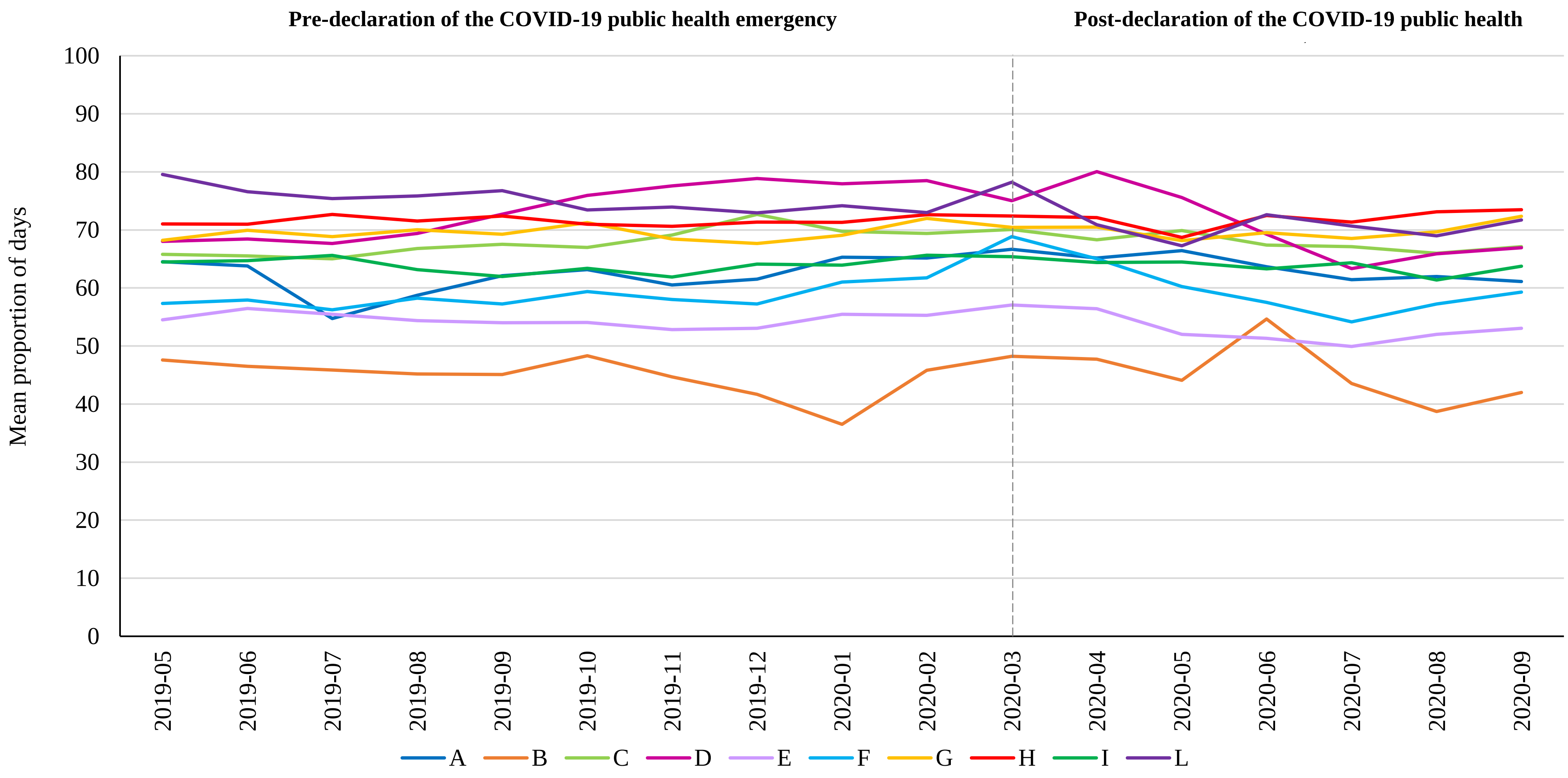

[a]Non-dual full benefit Medicaid enrollees ages 18-64 years in Kentucky, Maine, Maryland, Michigan, North Carolina, Ohio, Pennsylvania, Virginia, West Virginia, Wisconsin

© 2023 Austin AE et al. *JAMA Health Forum.*

eFigure 9. Monthly Rates of Any Medication for Opioid Use Disorder (MOUD) per 100,000 Medicaid Enrollees by Age Group[a]

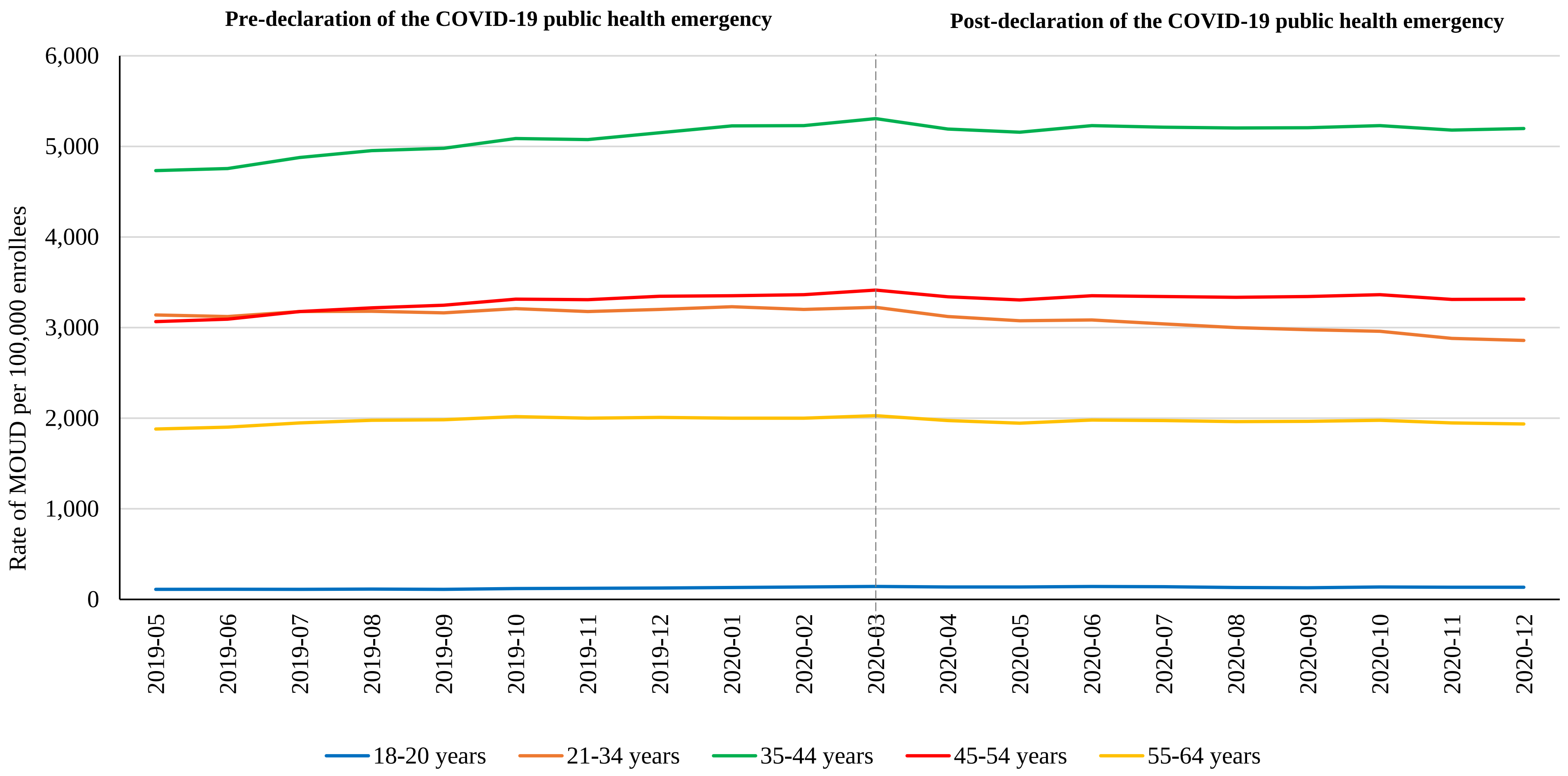

[a]Non-dual full benefit Medicaid enrollees ages 18-64 years in Kentucky, Maine, Maryland, Michigan, North Carolina, Ohio, Pennsylvania, Virginia, West Virginia, Wisconsin

© 2023 Austin AE et al. *JAMA Health Forum.*

eFigure 10. Monthly Rates of Outpatient Initiation[a] of Medication for Opioid Use Disorder (MOUD) per 100,000 Medicaid Enrollees by Age Group[b]

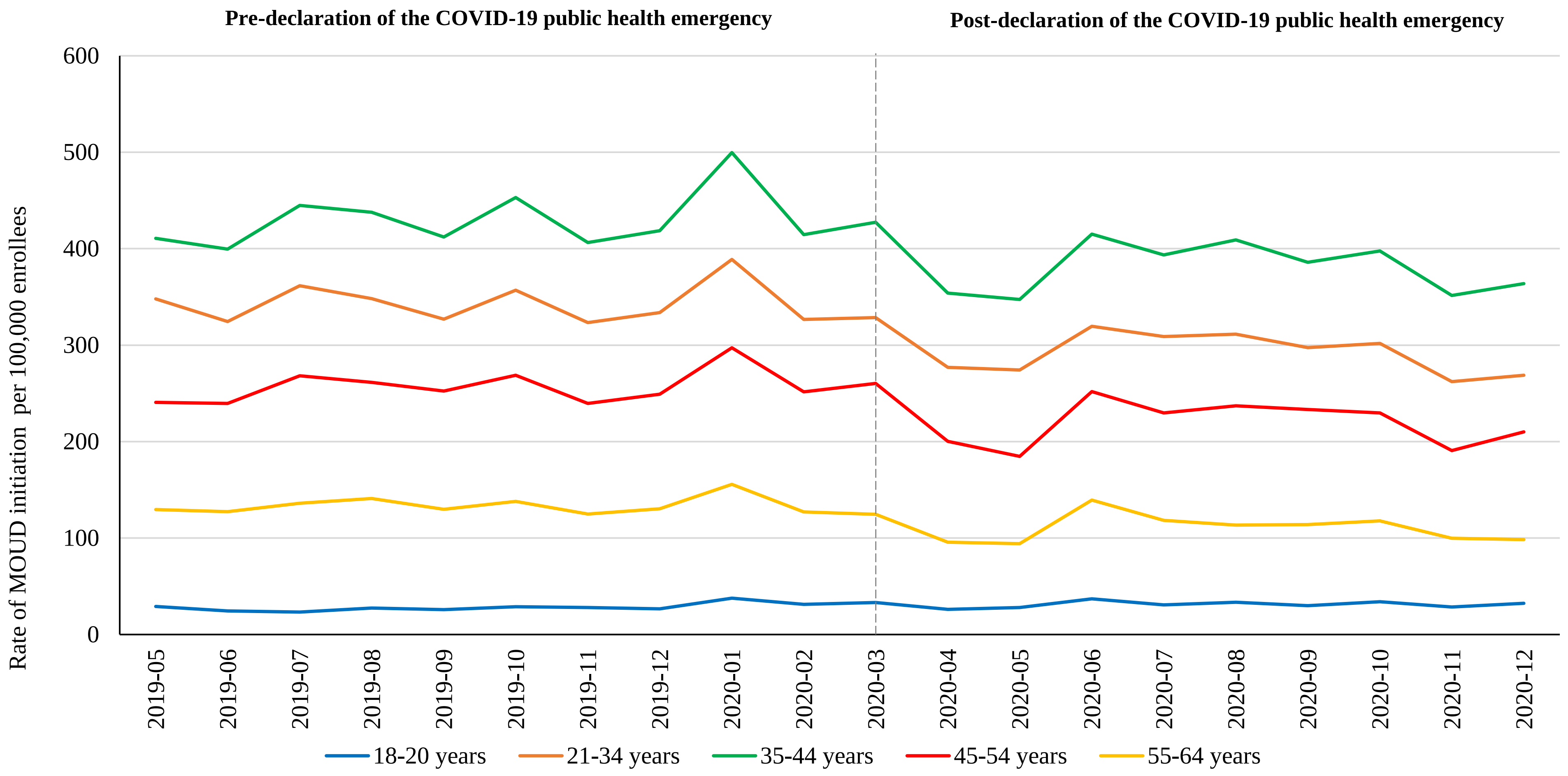

[a]Initiation defined as a new MOUD treatment episode following 30 days with no MOUD. All office- and facility-based MOUD administrations were classified as in-person initiations. Telehealth vs. outpatient initiation for prescription fills was determined by identifying outpatient visits within 7 days before to 3 days after the prescription fill with either a matching prescriber ID for the prescription and provider ID for the visit or with an opioid use disorder diagnosis at the visit and then applying state-specific definitions for telehealth. MOUD prescription fills with no outpatient visits within 7 days before to 3 days after the prescription fill meeting these criteria were unclassified.

[b]Non-dual full benefit Medicaid enrollees ages 18-64 years in Kentucky, Maine, Maryland, Michigan, North Carolina, Ohio, Pennsylvania, Virginia, West Virginia, Wisconsin

© 2023 Austin AE et al. *JAMA Health Forum.*

eFigure 11. Monthly Rates of Any Medication for Opioid Use Disorder (MOUD) per 100,000 Medicaid Enrollees by Race and Ethnicity[a]

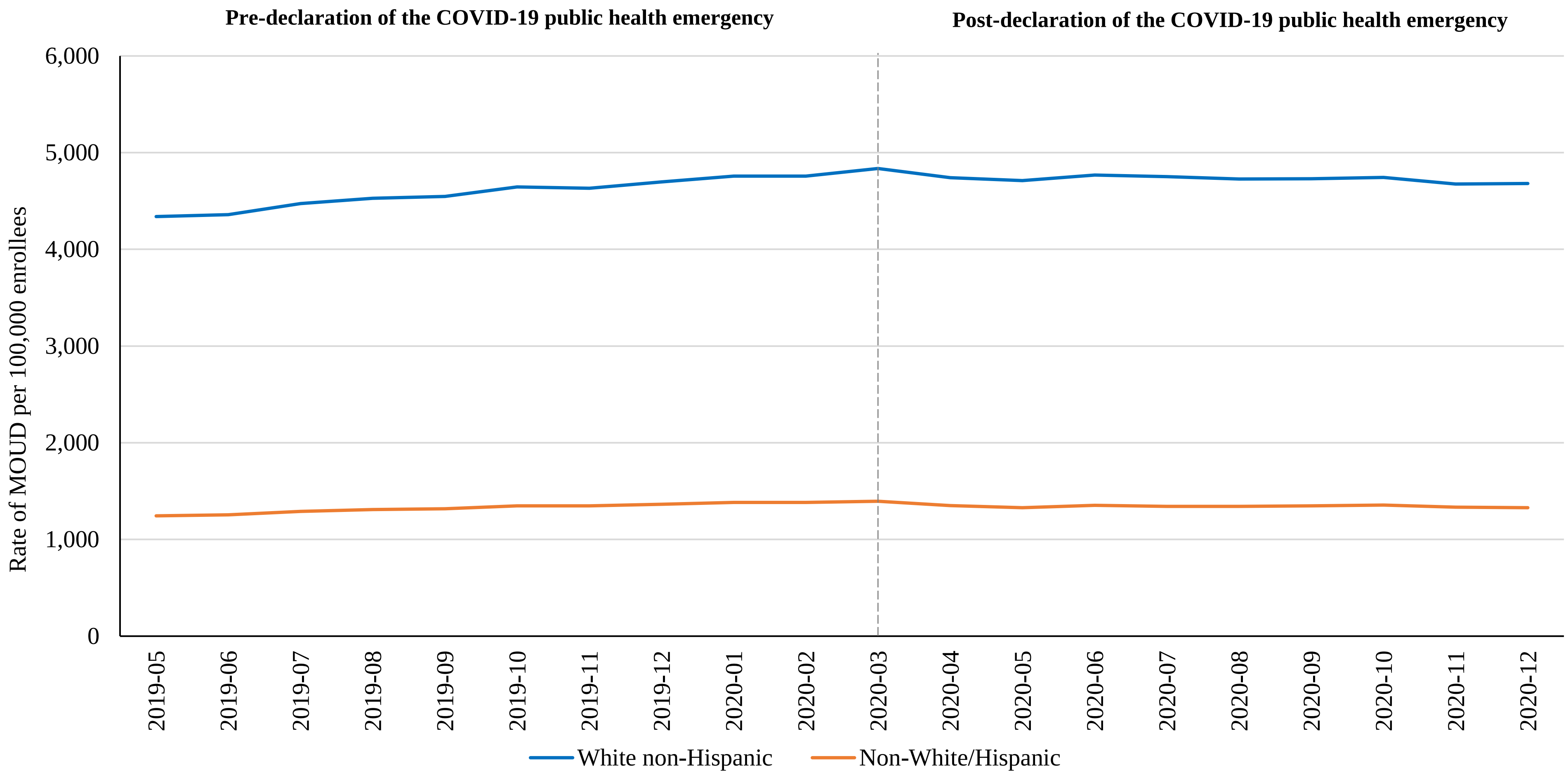

[a]Non-dual full benefit Medicaid enrollees ages 18-64 years in Kentucky, Maine, Maryland, Michigan, North Carolina, Ohio, Pennsylvania, Virginia, West Virginia, Wisconsin

© 2023 Austin AE et al. *JAMA Health Forum.*

eFigure 12. Monthly Rates of Outpatient Initiation[a] of Medication for Opioid Use Disorder (MOUD) per 100,000 Medicaid Enrollees by Race and Ethnicity[b]

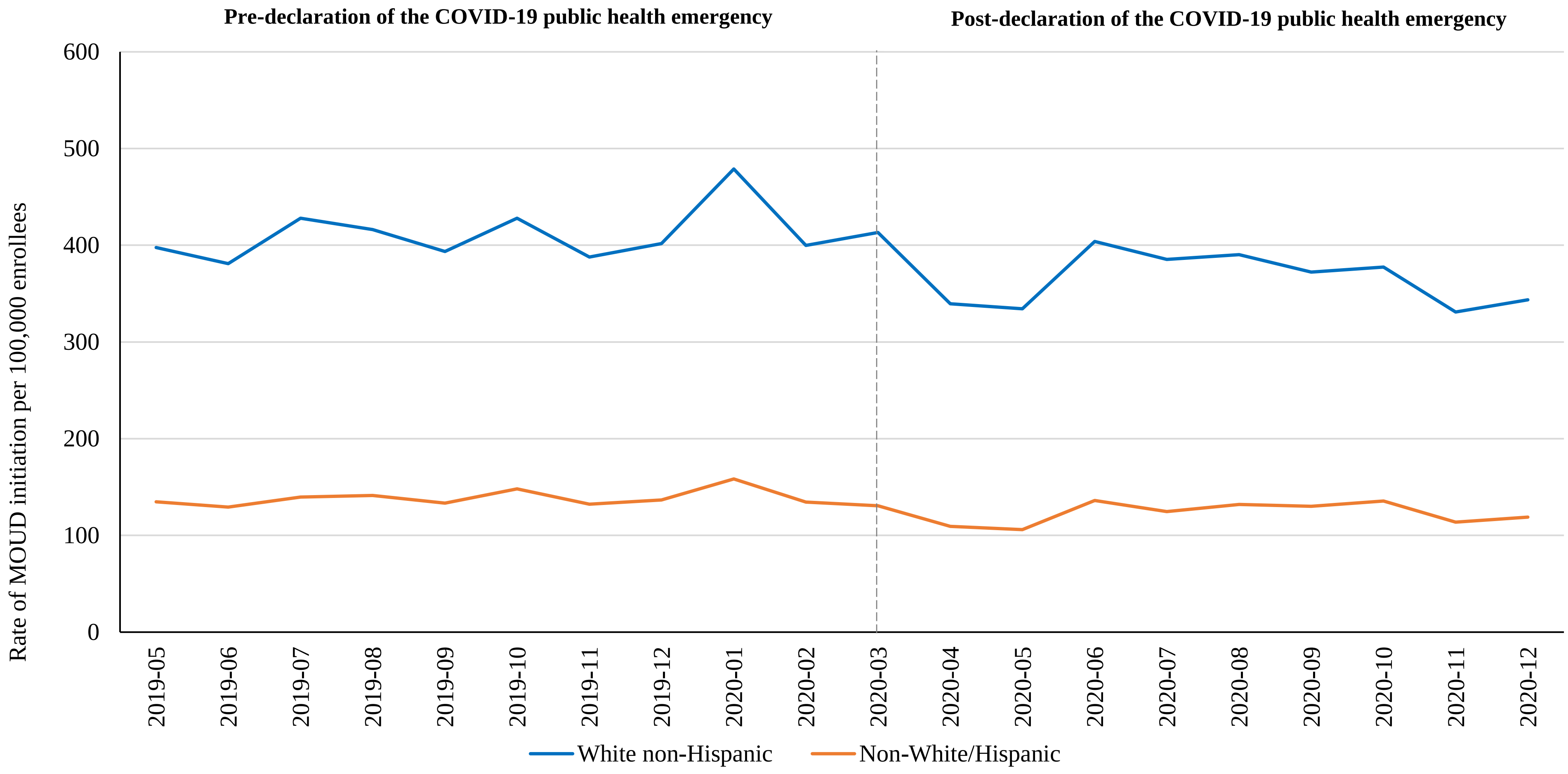

[a]Initiation defined as a new MOUD treatment episode following 30 days with no MOUD. All office- and facility-based MOUD administrations were classified as in-person initiations. Telehealth vs. outpatient initiation for prescription fills was determined by identifying outpatient visits within 7 days before to 3 days after the prescription fill with either a matching prescriber ID for the prescription and provider ID for the visit or with an opioid use disorder diagnosis at the visit and then applying state-specific definitions for telehealth. MOUD prescription fills with no outpatient visits within 7 days before to 3 days after the prescription fill meeting these criteria were unclassified.
[b]Non-dual full benefit Medicaid enrollees ages 18-64 years in Kentucky, Maine, Maryland, Michigan, North Carolina, Ohio, Pennsylvania, Virginia, West Virginia, Wisconsin

© 2023 Austin AE et al. *JAMA Health Forum.*

eFigure 13. Monthly Rates of Any Medication for Opioid Use Disorder (MOUD) per 100,000 Medicaid Enrollees by Sex[a]

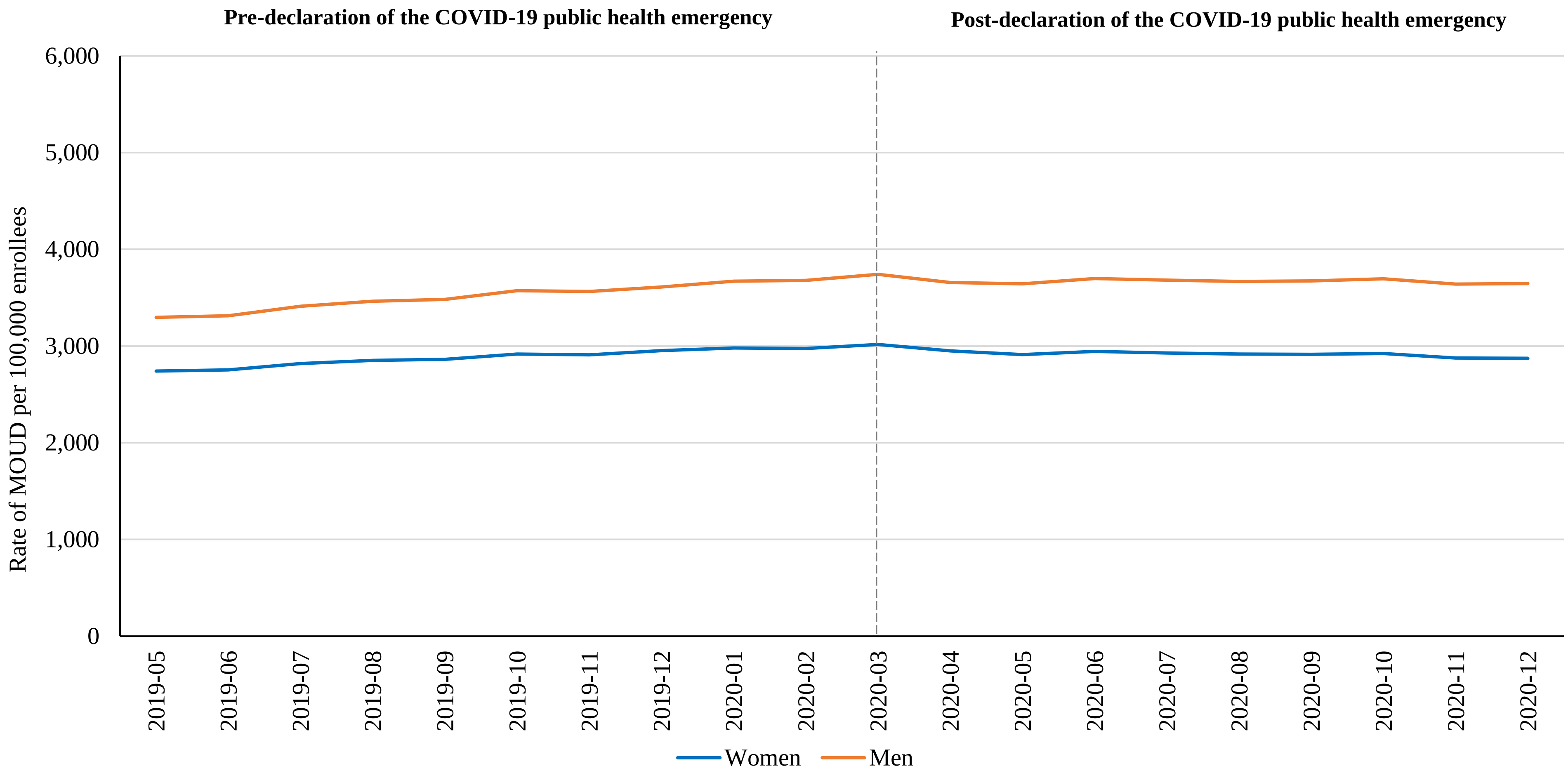

[a]Non-dual full benefit Medicaid enrollees ages 18-64 years in Kentucky, Maine, Maryland, Michigan, North Carolina, Ohio, Pennsylvania, Virginia, West Virginia, Wisconsin

© 2023 Austin AE et al. *JAMA Health Forum.*

eFigure 14. Monthly Rates of Outpatient Initiation[a] of Medication for Opioid Use Disorder (MOUD) per 100,000 Medicaid Enrollees by Sex[b]

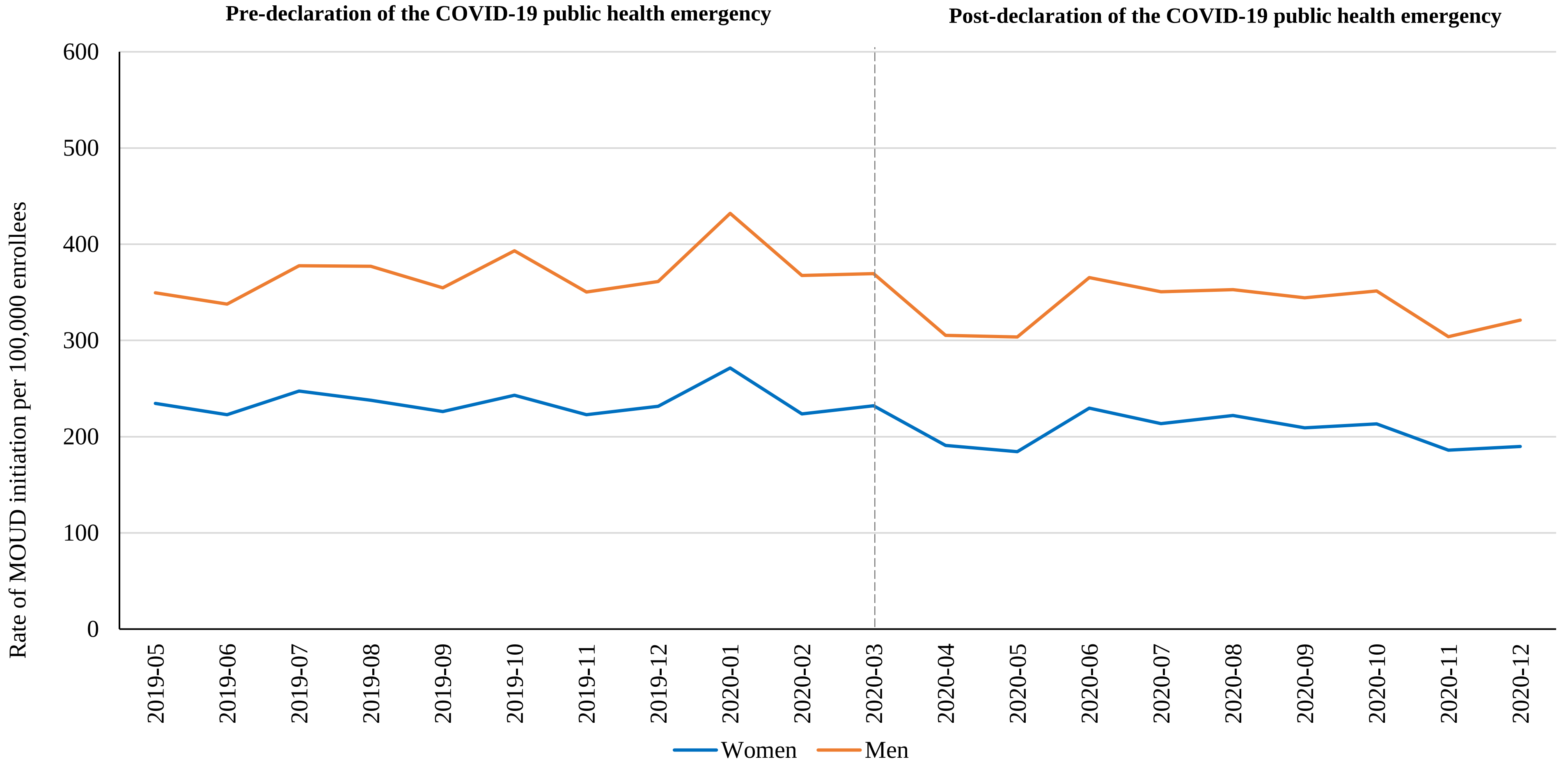

[a]Initiation defined as a new MOUD treatment episode following 30 days with no MOUD. All office- and facility-based MOUD administrations were classified as in-person initiations. Telehealth vs. outpatient initiation for prescription fills was determined by identifying outpatient visits within 7 days before to 3 days after the prescription fill with either a matching prescriber ID for the prescription and provider ID for the visit or with an opioid use disorder diagnosis at the visit and then applying state-specific definitions for telehealth. MOUD prescription fills with no outpatient visits within 7 days before to 3 days after the prescription fill meeting these criteria were unclassified.
[b]Non-dual full benefit Medicaid enrollees ages 18-64 years in Kentucky, Maine, Maryland, Michigan, North Carolina, Ohio, Pennsylvania, Virginia, West Virginia, Wisconsin

© 2023 Austin AE et al. *JAMA Health Forum.*

eFigure 15. Monthly Rates of Any Medication for Opioid Use Disorder (MOUD) per 100,000 Medicaid Enrollees by Living Area[a]

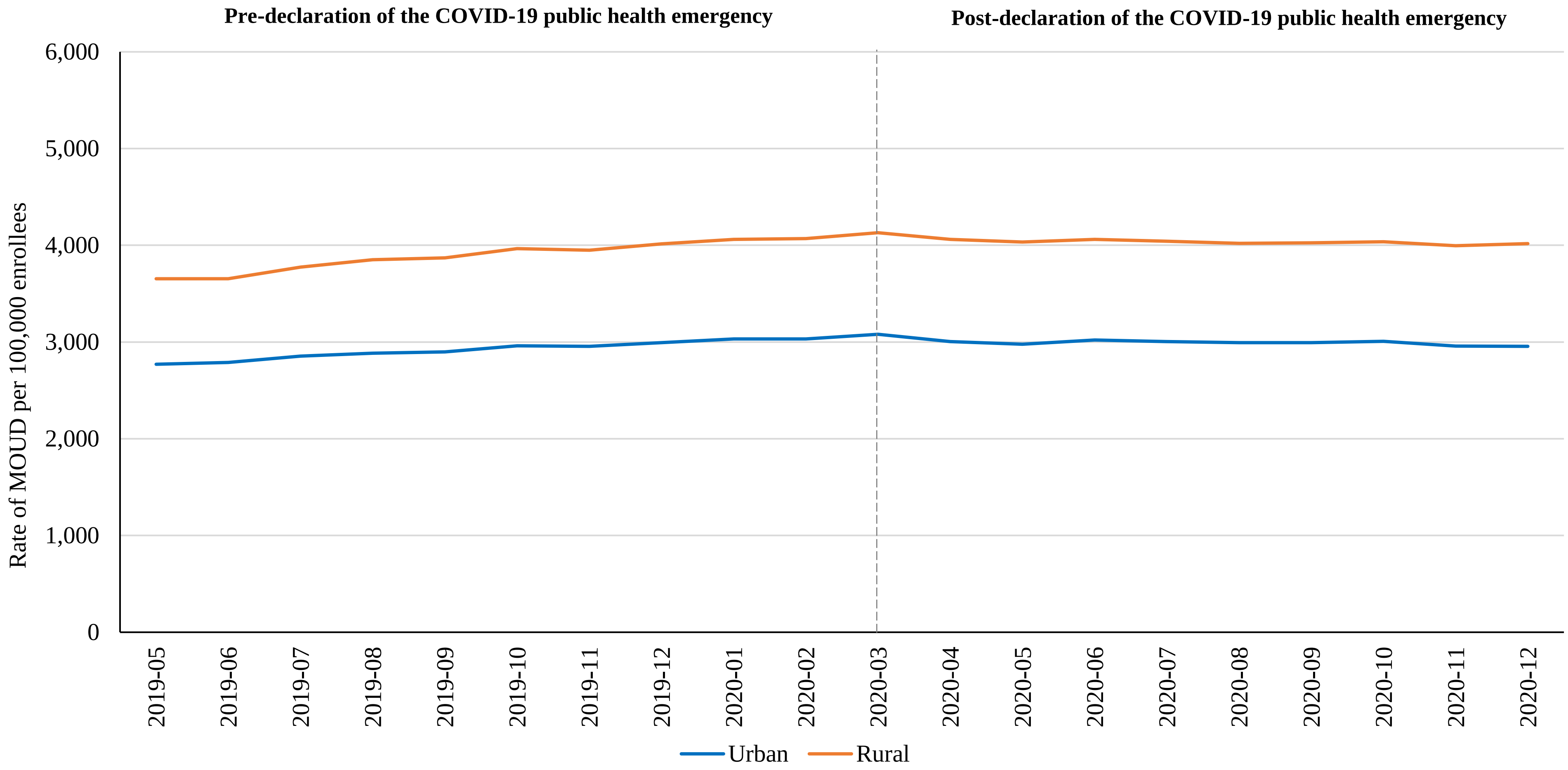

[a]Non-dual full benefit Medicaid enrollees ages 18-64 years in Kentucky, Maine, Maryland, Michigan, North Carolina, Ohio, Pennsylvania, Virginia, West Virginia, Wisconsin

© 2023 Austin AE et al. *JAMA Health Forum.*

eFigure 16. Monthly Rates of Outpatient Initiation[a] of Medication for Opioid Use Disorder (MOUD) per 100,000 Medicaid Enrollees by Living Area[b]

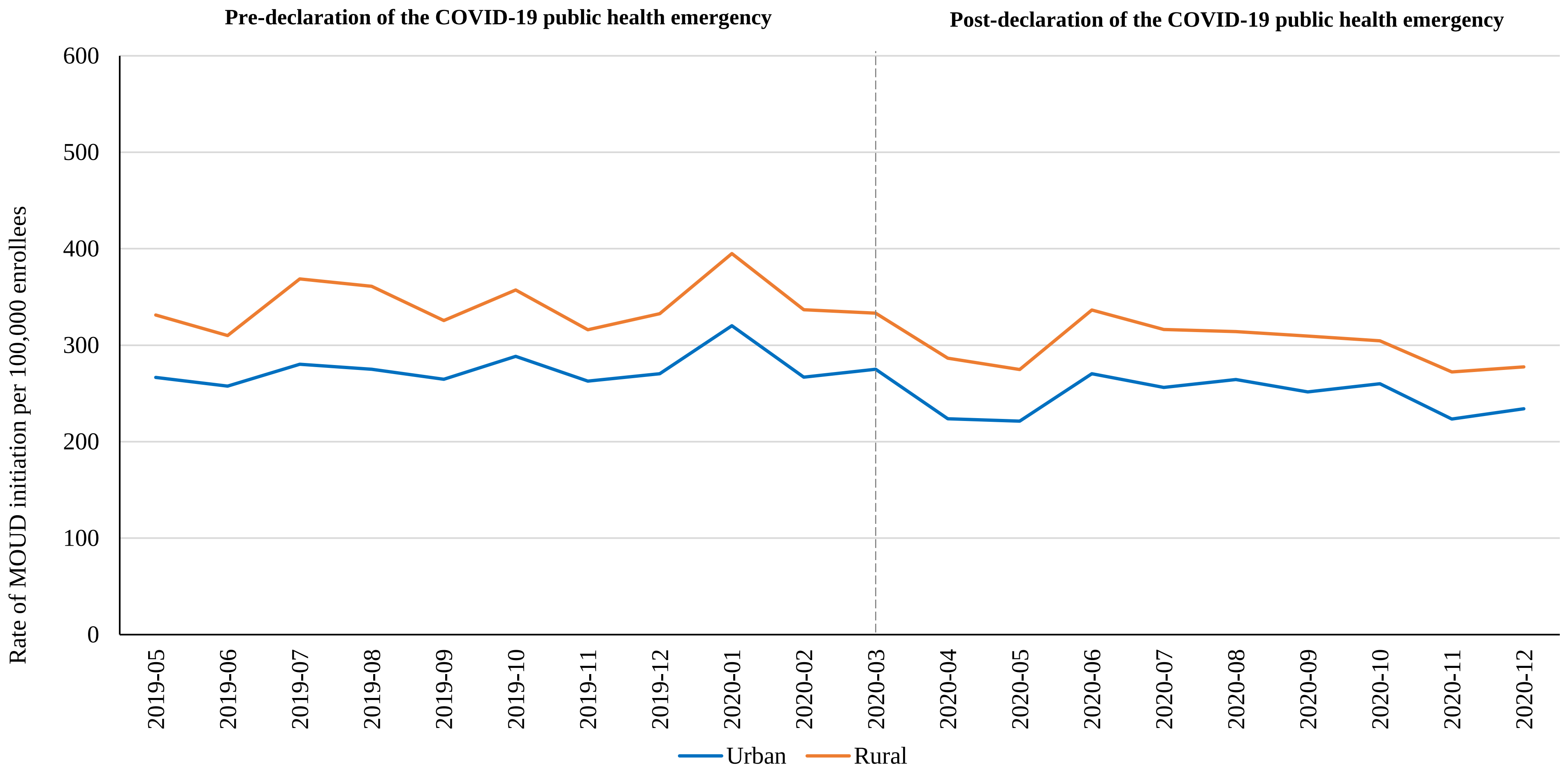

[a]Initiation defined as a new MOUD treatment episode following 30 days with no MOUD. All office- and facility-based MOUD administrations were classified as in-person initiations. Telehealth vs. outpatient initiation for prescription fills was determined by identifying outpatient visits within 7 days before to 3 days after the prescription fill with either a matching prescriber ID for the prescription and provider ID for the visit or with an opioid use disorder diagnosis at the visit and then applying state-specific definitions for telehealth. MOUD prescription fills with no outpatient visits within 7 days before to 3 days after the prescription fill meeting these criteria were unclassified.

[b]Non-dual full benefit Medicaid enrollees ages 18-64 years in Kentucky, Maine, Maryland, Michigan, North Carolina, Ohio, Pennsylvania, Virginia, West Virginia, Wisconsin

© 2023 Austin AE et al. *JAMA Health Forum.*

eTable 3. State-Specific Interrupted Time Series Results[a] for Change in the Likelihood of Any Medication for Opioid Use Disorder (MOUD) After the COVID-19 Public Health Emergency Was Declared (Post-PHE) Compared With Before the Public Health Emergency Was Declared (Pre-PHE)[b] Among Medicaid Enrollees in 10 States[c]

	**Time trend**		**Immediate change after vs before the PHE**		**Trend change after vs before the PHE**	
State	OR	95% CI	OR	95% CI	OR	95% CI
A	1.02	(1.02,1.02)	1.01	(1.00, 1.01)	1.00	(1.00, 1.00)
B	0.99	(0.99,1.00)	1.00	(1.00, 1.01)	1.01	(1.01, 1.01)
C	1.01	(1.01, 1.01)	1.00	(1.00, 1.01)	1.00	(1.00, 1.00)
D	1.00	(1.00, 1.00)	1.01	(1.00, 1.01)	1.01	(1.01, 1.01)
E	1.00	(1.00, 1.01)	1.01	(1.00, 1.01)	1.00	(1.00, 1.00)
F	1.00	(1.00, 1.00)	1.02	(1.02,1.03)	1.00	(1.00, 1.00)
G	1.03	(1.03,1.03)	1.01	(1.00, 1.02)	0.99	(0.99,0.99)
H	1.00	(1.00, 1.01)	1.00	(0.99,1.01)	1.00	(1.00, 1.00)
I	1.01	(1.01, 1.01)	1.00	(0.99,1.01)	1.01	(1.00, 1.01)
L	1.01	(1.00, 1.01)	1.01	(1.00, 1.02)	1.00	(0.99, 1.00)

OR=odds ratio; CI=confidence interval

[a]Adjusted for sex, race/ethnicity, age group, area of residence, Medicaid eligibility group, and number of months enrolled in Medicaid during the study period

[b]Post-PHE period is March-December 2020. Pre-PHE period is May 2019-February 2020.

[c]Non-dual full benefit Medicaid enrollees ages 18-64 years in Kentucky, Maine, Maryland, Michigan, North Carolina, Ohio, Pennsylvania, Virginia, West Virginia, Wisconsin

© 2023 Austin AE et al. *JAMA Health Forum.*

eTable 4. State-Specific Interrupted Time Series Results[a] for Change in the Likelihood of Outpatient Initiation of Medication for Opioid Use Disorder (MOUD) After the COVID-19 Public Health Emergency Was Declared (Post-PHE) Compared With Before the Public Health Emergency Was Declared (Pre-PHE)[b] Among Medicaid Enrollees in 10 States[c]

	**Time trend**		**Immediate change after vs before the PHE**		**Trend change after vs before the PHE**	
State	OR	95% CI	OR	95% CI	OR	95% CI
A	1.02	(1.02,1.03)	0.89	(0.86,0.91)	0.97	(0.96,0.97)
B	1.01	(1.00,1.01)	0.91	(0.88,0.95)	1.00	(0.99,1.00)
C	1.01	(1.01,1.02)	0.75	(0.72,0.78)	0.99	(0.98,1.00)
D	1.00	(0.99,1.01)	1.05	(0.99,1.11)	0.99	(0.98,1.00)
E	1.00	(1.00,1.01)	0.93	(0.90,0.95)	0.99	(0.99,1.00)
F	1.00	(1.00,1.00)	0.92	(0.89,0.94)	0.99	(0.99,1.00)
G	1.00	(1.00,1.01)	0.86	(0.82,0.89)	0.99	(0.99,1.00)
H	1.00	(0.99,1.00)	0.97	(0.92,1.03)	0.98	(0.97,0.99)
I	1.01	(1.00,1.02)	0.91	(0.87,0.96)	1.00	(0.99,1.01)
L	0.99	(0.98,1.01)	0.89	(0.81,0.98)	0.97	(0.95,0.99)

OR=odds ratio; CI=confidence interval

[a]Adjusted for sex, race/ethnicity, age group, area of residence, Medicaid eligibility group, and number of months enrolled in Medicaid during the study period

[b]Post-PHE period is March-December 2020. Pre-PHE period is May 2019-February 2020.

[c]Non-dual full benefit Medicaid enrollees ages 18-64 years in Kentucky, Maine, Maryland, Michigan, North Carolina, Ohio, Pennsylvania, Virginia, West Virginia, Wisconsin

© 2023 Austin AE et al. *JAMA Health Forum.*

eTable 5. MODRN Collaborators

Delaware: Elizabeth Brown, MD, MSHP, Dara Hall, MSN, RNC-NIC (Delaware Division of Medicaid and Medical Assistance)

Kentucky: Katherine Marks, PhD (Kentucky Department for Behavioral Health, Developmental and Intellectual Disabilities), Angela Taylor, BS (University of Kentucky and Kentucky Office of Health Data Analytics)

Maine: Catherine McGuire, BS (University of Southern Maine), David Jorgenson, MS (Maine Department of Health and Human Services)

Maryland: Alyssa Brown, JD (Maryland Department of Health), Alice Middleton, JD, Cynthia Woodcock, MBA, David Idala, MA (The Hilltop Institute, UMBC)

Michigan: Lisa Cohn, MS (University of Michigan, Department of Pediatrics), Marie LaPres, RN, JD (Michigan Medicaid)

North Carolina: Emma Sandoe, PhD, Shannon Dowler, MD, Lauryn Walker, PhD, RN, MPH (North Carolina Department of Health and Human Services)

Ohio: Mary Applegate, MD, FAAP, FACP, Kendallyn Markman, Mark Rizzutti, MPH, MHSA, Elizabeth Truex-Powell, PhD (Ohio Department of Medicaid), Aimee Mack, MPH, Robert Ashmead, PhD, Emelie Bailey, MA (Ohio Colleges of Medicine Government Resource Center, Ohio State University Wexner Medical Center)

Pennsylvania: David Kelley, MD, Andrea Race, (Pennsylvania Department of Human Services), A. Everette James, JD, MBA, Monica Costlow, JD (University of Pittsburgh)

Virginia: Ashley Harrell, LCSW, Neil McCray, PhD, Chethan Bachireddy, MD, MSc ((Virginia Department of Medical Assistance Services)

West Virginia: Cynthia Parsons, MA, James Becker, MD (Bureau for Medical Services at the West Virginia Department for Health and Human Resources), Yilin Cai, MS, MBA (West Virginia University)

Wisconsin: Kristen Voskuil, MA (University of Wisconsin), Pam Appleby (Wisconsin Department of Health Services)

© 2023 Austin AE et al. *JAMA Health Forum.*
