# Supplementary material for: Trends in Use of Medication to Treat Opioid Use Disorder During the COVID-19 Pandemic in 10 State Medicaid Programs
Source: JAMA Health Forum. 2023 Jun 16;4(6):e231422. doi: 10.1001/jamahealthforum.2023.1422 (PMC10276306; doi:10.1001/jamahealthforum.2023.1422)
Supplement: Supplement 2. — Data Sharing Statement [file jamahealthforum-e231422-s002.pdf]

Data Sharing Statement

Austin. Trends in Use of Medication to Treat Opioid Use Disorder During the COVID-19 Pandemic in 10 State Medicaid Programs. *JAMA Health Forum*. Published June 16, 2023. doi:10.1001/jamahealthforum.2023.1422

Data
Data available: No

Additional Information
Explanation for why data not available: Medicaid claims and enrollment data were available to University partners by their state Medicaid agency through data use agreements.
